# Methodological quality of systematic intervention reviews on vaccination

**DOI:** 10.1186/s13643-025-03052-2

**Published:** 2026-01-07

**Authors:** Antonia Pilic, Louise Henaff, Christoph A. Steffen, Madeleine Batke, Hanna Helene Linß, Iksha Thebe, Nina Rehr, Catalina Hamon Pinilla, Amrita John, Ole Wichmann, Vanessa Piechotta, Thomas Harder

**Affiliations:** 1 Robert Koch Institute, Immunization Unit, Berlin, Germany; 2 Charité-Universitätsmedizin Berlin, Corporate Member of Freie Universität Berlin and Humboldt-Universität Zu Berlin, Berlin, Germany; 3 World Health Organization (WHO), 20 Avenue Appia, Geneva, 1211 Switzerland

**Keywords:** Methodological quality, AMSTAR 2, Systematic review, National Immunization Technical Advisory Group (NITAG), Methodology, Vaccination

## Abstract

**Background:**

Systematic reviews (SRs) are pivotal in vaccination recommendation development. However, the reliability of their findings relies on methodological rigor. This study examines the methodological quality of vaccination-related SRs and aims to identify influencing factors.

**Methods:**

We used the SYSVAC registry to draw 120 SRs on the efficacy or effectiveness of vaccination using a randomized block design. SRs published from 2011 to 2023 were considered. SR characteristics were extracted, and methodological quality was assessed by two reviewers using AMSTAR 2 (A Measurement Tool to Assess Systematic Reviews 2). SRs were scored between 0 and 16 and categorized as high, moderate, low, or critically low quality. Mann–Whitney U test, chi-squared test, Fisher’s exact test, and multivariable linear regression analysis were used to assess the influence of SR characteristics on AMSTAR 2 score. Secondary analysis of critically low-rated SRs was conducted to identify limitations in critical items.

**Results:**

Out of 120 SRs, 110 SRs were rated as of critically low-quality. The majority of critically low-rated SRs lacked the justification for excluding individual studies (*n* = 103, 93.6%) and protocol registration (*n* = 85, 77.3%). Median AMSTAR 2 score across all SRs was 10 (range 2–16). SRs published after 2017, Cochrane reviews, and SRs from authors with no conflicts of interest (CoI) had higher methodological quality than those published before 2017, non-Cochrane reviews, and SRs from authors with CoI (mean difference AMSTAR 2 score 2, 6, and 2, respectively; all *p* < 0.05). SRs published before 2017 had significant limitations in protocol registration, study selection in duplicate, and risk of bias assessment; and non-Cochrane reviews in protocol registration, justification for excluding individual studies, funding sources of studies, and impact of risk of bias assessment of individual studies (all *p* < 0.05). Management of conflicts was described only in a quarter of SRs with CoI (*n* = 9/43, 20.9%). The adjusted analysis showed that only publication year after 2017 and Cochrane review status had a significant independent relation on the AMSTAR 2 score (R^2^ = 0.26; adjusted R^2^ = 0.17).

**Conclusion:**

The findings underscore the critical methodological shortcomings in vaccination-related SRs. Future efforts should prioritize adherence to established methodological standards and transparency, to enhance the impact of SRs in individual and decision-making.

**Supplementary Information:**

The online version contains supplementary material available at 10.1186/s13643-025-03052-2.

## Background

Critical assessment of the methodological quality of systematic reviews (SRs) is crucial. Among several tools available to assess the quality of SRs [1–3], AMSTAR 2 (A Measurement Tool to Assess Systematic Reviews 2) is one of the most often used [2, 4]. AMSTAR 2 was developed in 2017 for SRs of randomized and non-randomized controlled trials of healthcare interventions [2]. The tool consists of 16 items which have to be answered using four response categories (yes/partial yes/no/no meta-analysis conducted) [2]. A higher number of fulfilled items (i.e., answered by “yes”) corresponds to a higher methodological quality, ranging from a score of 0 to 16. Seven items are defined as being of critical importance. Based on identified flaws in items, AMSTAR 2 classifies the overall confidence of a SR into four categories: high, moderate, low, and critically low.

National Immunization Technical Advisory Groups (NITAGs) are independent expert groups which develop vaccination recommendations for national immunization programs [5]. Regarding efficacy, effectiveness, and safety of vaccines, NITAGs are requested to base their recommendations on best-available evidence, ideally the results of SRs. A recent bibliometric analysis showed a substantial increase in the number of vaccination-related SRs over the past decade, with a major proportion of them being rated as critically low in overall methodological quality [6]. However, no detailed analysis on the individual AMSTAR 2 items has been conducted in this study [6]. Using reliable and transparently reported SRs without methodological flaws is crucial for NITAGs to develop evidence-based vaccination recommendations, reducing the risk of false conclusions that could negatively impact decision-making and trust in the recommendation.

Therefore, the aim of this study was (i) to assess the methodological quality of SRs on the efficacy and effectiveness of vaccines and (ii) to investigate the characteristics influencing the methodological quality of these SRs.

## Methods

We performed a cross-sectional study following a protocol developed a priori (see Additional file 1).

### Database and review selection

We used the SYSVAC registry to draw a sample of SRs on the efficacy or effectiveness of vaccination. SYSVAC is a freely accessible database hosted by the NITAG Resource Center [7], which has its secretariat based at the World Health Organization (see www.nitag-resource.org/sysvac-systematic-reviews) [8]. As of 15 August 2024, SYSVAC comprised a total of 2275 SRs (including umbrella reviews) on vaccination-related topics. SRs included in SYSVAC are identified by monthly systematic literature searches conducted in MEDLINE on the OVID platform, Embase, the Cochrane Library of Systematic Reviews, and the Living Overview of Evidence repository [9] (for details, see Additional file 2 and [8, 10]). Outcomes considered in SYSVAC include microbiological, biological, or immunological markers (e.g., antibodies, immune cells), along with epidemiological factors (e.g., infection) and the impact on disease epidemiology (including indirect effects and impact on serotype distribution). We used block randomization to draw a sample of 120 SRs from SYSVAC. Blocks were defined according to a single vaccine-preventable disease being the topic of the respective SR, as follows: block 1: human papillomavirus (HPV), block 2: influenza, and block 3: other vaccine-preventable diseases. Thereby, each block included 40 SRs. Only SRs published between 2011 and 2023 were considered to focus on current trends and standards. The SYSVAC registry was initiated in 2011 and only SRs published from this time onwards are incorporated in the registry. The incorporation of SRs published in 2024 had not been completed at the time point of the analysis. All targeted populations and settings were eligible. Umbrella reviews were not considered.

### Data extraction and methodological quality assessment

Two reviewers (AP, HL, MB, IT, NR, CP, AJ) extracted the following SR characteristics from the SRs into an Excel extraction form: year of publication; disease/pathogen focus of SR; number of authors; institution and country of corresponding author; Cochrane review; type of included studies in SR; funding information of SR; and declaration of conflicts of interest (CoI). The methodological quality was assessed by pairs of experienced SR methodologists (AP, HL, MB, IT, NR, CP) using AMSTAR 2 (published first in 2017 [2]), with minor adjustments of some items against the original tool for facilitated analysis (see Table 1 for further details). All 16 items were assessed, and judgments were justified by quotes. Each item was answered with either “yes”, “partial yes”, “no” or “no meta-analysis conducted” (if appropriate). Any disagreements where no guidance was available from the AMSTAR 2 tool guidance (e.g., item 16 on dealing with conflicts of interests) were solved through discussion until consensus was achieved. A summary score was calculated by summing the number of items fulfilled (i.e., answered by “yes”, “partial yes”, and “no meta-analysis conducted”). According to AMSTAR 2, high-quality denotes no or one non-critical weakness identified; moderate-quality denotes more than one non-critical weakness identified; low-quality denotes one critical flaw with or without non-critical weaknesses identified; and critically low-quality denotes more than one critical flaw with or without non-critical weaknesses identified (see Table 1 for critical items) [2].

**Table 1:** Description of original AMSTAR 2 items and adjustments

Item	Description of original AMSTAR 2 item [2]	Adjustment
1	Did the research question and inclusion criteria for the review include the components of PICO?	-
**2**	**Was there “a priori” protocol designed and any significant deviations from the protocol justified?**	Only “yes” or “no” option as generally binary outcome
3	Was the selection of the study designs for inclusion in the review explained?	Any information on study type sufficient to meet this item
**4**	**Was a comprehensive literature search strategy used? (subitem: justified publication restrictions)**	Any information on restrictions sufficient to meet subitem
5	Was study selection performed in duplicate?	-
6	Was data extraction performed in duplicate?	-
**7**	**Did the review authors provide a list of excluded studies and justify the exclusions?**	Only “yes” or “no” option as generally binary outcome
8	Were the included studies described in adequate detail?	Only “yes” or “no” option as generally binary outcome
**9**	**Was a satisfactory technique used for assessing the RoB in individual studies included in the review?**	Only “yes” or “no” option as generally binary outcome
10	Were sources of funding for the studies included in the review reported?	-
**11**	Were appropriate methods for statistical combination of results used?*	-
12	Was the potential impact of RoB in individual studies on the results assessed?*	-
**13**	**Did the review authors account for RoB in individual studies when interpreting/discussing the results of the review?**	-
14	Was a satisfactory explanation for, and discussion of any heterogeneity observed in the results provided?	-
**15**	Was an adequate investigation on publication bias conducted and its likely impact on the results discussed?*	-
16	Were potential conflicts of interest declared and how were they dealt with?	-

Items in bold are critical items

^*^if meta-analysis conducted (term “meta-analysis” needs to be mentioned)

*PICO* Population, Intervention, Comparator, Outcome, *RoB* Risk of bias

### Statistical analysis

Descriptive statistics are displayed as median and range or frequencies and percentages, as appropriate. Differences in AMSTAR 2 score according to SR characteristics were compared using Mann–Whitney U test. Chi-squared test or Fisher’s exact test was used to compare individual AMSTAR 2 items. Multivariable linear regression was applied for influence analysis of SR characteristics on AMSTAR 2 score. Secondary analysis was conducted for SRs rated as critically low to identify limiting patterns of critical items (items 2, 4, 7, 9, 11, 13, and 15) that contributed to their AMSTAR 2 rating. For all analyses, the responses “yes”, “partial yes” and “no meta-analysis conducted” were combined into one response, and the item was considered to be fulfilled. *P*-values below 0.05 were considered statistically significant. All statistical analyses were carried out using R (version 4.4.1).

## Results

**Fig. 1:**
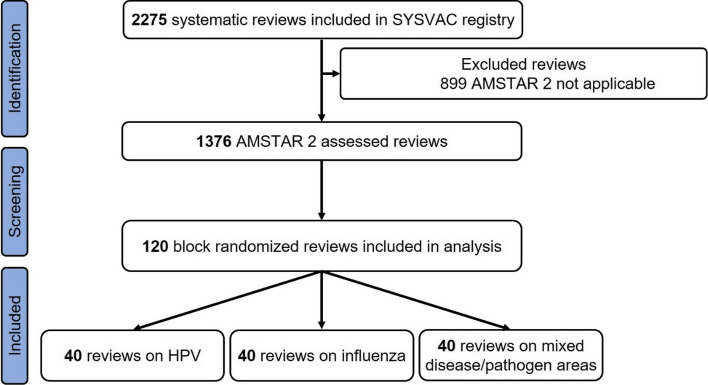
Flow chart of block-randomized selection process for systematic reviews on vaccination efficacy/effectiveness included in SYSVAC registry [8] *AMSTAR 2* A Measurement Tool to Assess Systematic Reviews 2, *HPV* Human papillomavirus

The search in the SYSVAC registry identified 2275 SRs, of which 1376 SRs addressed interventions (i.e., efficacy or effectiveness of vaccines) and were therefore eligible for AMSTAR 2. According to study design, 120 SRs were included in the analysis (see Fig. 1 for flow chart). A list of the SRs not considered in this analysis is available upon request.

About half of the included SRs were published in or after 2019 and had a median of five authors. Most SRs had a corresponding author with an academic background (*n* = 64; 53.3%), followed by a background in policy/government (*n* = 20; 16.7%). Three SRs were Cochrane reviews. The majority of SRs included randomized-controlled trials (RCTs; *n* = 87, 72.5%). Out of the 87 SRs with reporting on funding, 18 SRs (15.0%) were funded by pharmaceutical companies. Forty-three SRs (35.8%) reported that at least one author had a CoI. Table 2 summarizes further characteristics of the included SRs. Details on individual SRs are shown in Additional file 3.

**Table 2:** Characteristics of included vaccination-related systematic reviews. *N* = 120

Characteristics of systematic reviews	Median (range) or n (%)
Year of publication	2019 (2011–2023)
Disease/pathogen (intervention)	
HPV, Influenza (each)	40 (33.3)
Tuberculosis	5 (4.2)
Herpes zoster	4 (3.3)
Cholera, Dengue, HIB, Poliomyelitis, TBE, Varicella (each)	3 (2.5)
Hepatitis A, Yellow fever (each)	2 (1.7)
Diphtheria, Ebola, Herpes simplex, Meningococcal disease, Mpox, Mumps, Rabies, Streptococcus group B, Zika virus (each)	1 (0.8)
Number of authors	5 (1–18)
Institution of corresponding author	
Academia	64 (53.3)
Policy/government	20 (16.7)
Industry	18 (15.0)
Cross-sector	18 (15.0)
Country of corresponding author	
United States of America	21 (17.5)
China, United Kingdom (each)	11 (9.2)
Australia, Italy (each)	10 (8.3)
Canada	9 (7.5)
Switzerland	5 (4.2)
Brazil, Germany, Netherlands (each)	4 (3.3)
France	3 (2.5)
Bangladesh, Belgium, Denmark, Ireland, Republic of Korea, Peru, Portugal, South Africa, Spain, Taiwan (each)	2 (1.7)
Argentina, Guinea-Bissau, Hungary, India, Islamic Republic of Iran, Japan, Norway, Poland, Singapore, Sweden, Tanzania, Zambia (each)	1 (0.8)
Cochrane review	3 (2.5)
Study design included (multiple possible)	
RCTs	87 (72.5)
NRSIs	65 (54.2)
Single-arm studies	15 (12.5)
Funding by pharmaceutical company	
No	69 (57.5)
Not reported	33 (27.5)
Yes	18 (15.0)
Conflicts of interests declared	
No	74 (61.7)
Yes	43 (35.8)
Not reported	3 (2.5)

*HIB Haemophilus influenzae type b*, *HPV* Human papillomavirus, *NRSIs* Non-randomized studies of interventions, *RCTs* Randomized-controlled trials, *TBE* Tick-borne encephalitis

The methodological quality of most SRs was critically low, with flaws in more than one critical item (*n* = 110, 91.7%). Six SRs were of high quality (5.0%), of which four had flaws in one non-critical item (item 6, 10, 12, or 14). The median of the AMSTAR 2 score, including meta-analysis considering items, was 10 (range 2–16). Table 3 summarizes the methodological quality of the included SRs.

**Table 3:** Methodological quality of included vaccination-related systematic reviews. *N* = 120

Methodological quality of systematic reviews	Median (range) or n (%)
AMSTAR 2 overall confidence	
*Critically low*	110 (91.7)
*High*	6 (5.0)
*Low*	4 (3.3)
*Moderate*	0 (0.0)
AMSTAR 2 score	10 (2–16)
AMSTAR 2 score (excl. meta-analysis considering items 11, 12, and 15)	9 (0–16)

*AMSTAR 2* A Measurement Tool to Assess systematic Reviews 2

**Fig. 2:**
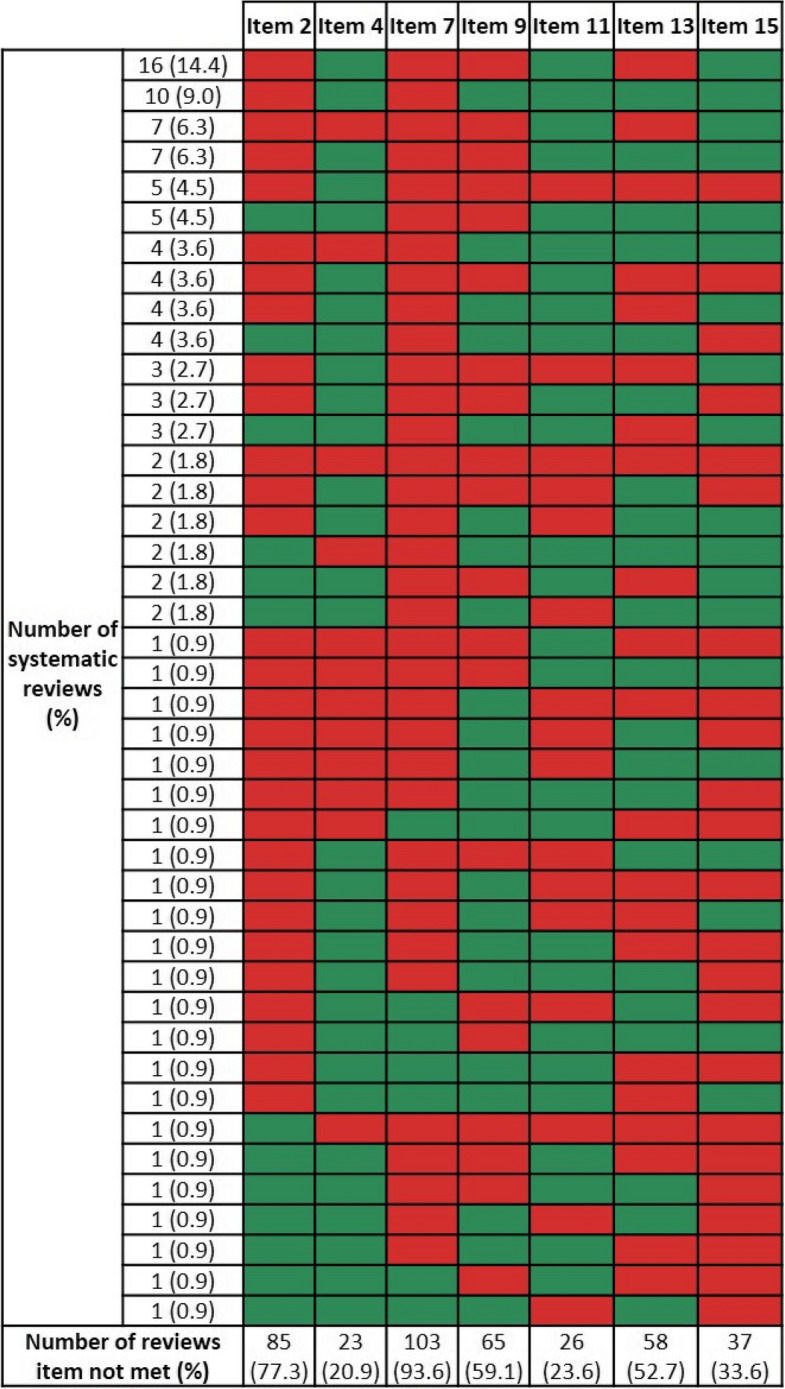
Patterns of AMSTAR 2’s limitations in critical items of systematic reviews. Red indicates limitations and green fulfillment of an item. *Item 2* Protocol registration, *Item* *4* Adequacy of literature search strategy, *Item* *7* Justification for excluding individual studies, *Item 9* Risk of bias assessment, *Item 11* Appropriateness of meta-analytical methods, *Item 13* Risk of bias interpretation, *Item 15* Publication bias. Further information on AMSTAR 2 critical items can be found in Table 1. *N* = 110

Secondary analysis of critically low-rated SRs (*n* = 110) showed that the majority of them failed to meet two to four out of seven critical items (see Table 4 for number of critical items not met). Almost all critically low-rated SRs lacked the justification for excluding individual studies (item 7; *n* = 103, 93.6%) and did not report a protocol registration (item 2; *n* = 85, 77.3%). Only some SRs did not report an adequate literature search strategy (item 4; *n* = 23, 20.9%) or applied appropriate meta-analytical methods (item 11; *n* = 26, 23.6%). Most frequent combinations of unmet critical items were lack of protocol registration (item 2) plus lack of justification for excluding individual studies (item 7) plus no or inadequate risk of bias (RoB) assessment (item 9) and interpretation (item 13) (*n* = 16, 14.4%); or the lack of protocol registration (item 2) plus justification for excluding individual studies (item 7) (*n* = 10, 9.0%) (Fig. 2). All AMSTAR 2 ratings can be found in Additional file 4.

**Table 4:** Limitations in AMSTAR 2’ critical items of *critically low*-rated systematic reviews

Number of limitations in critical items	Number of SRs (*n*)	%
Two	29	26.4
Three	25	22.7
Four	28	25.5
Five	18	16.4
Six	8	7.3
All seven	2	1.8

*Item 2* Protocol registration, *Item 4* Adequacy of literature search strategy, *Item 7* Justification for excluding individual studies, *Item 9* Risk of bias assessment, *Item 11* Appropriateness of meta-analytical methods, *Item 13* Risk of bias interpretation, *Item 15* Publication bias. Further information on AMSTAR 2 critical items can be found in Table 1 . *N *= 110

Bivariate analyses showed that SRs published after 2017 (AMSTAR 2 was published first in 2017 [2]) and Cochrane reviews had a significantly higher methodological quality than SRs published before 2017 (mean AMSTAR 2 score difference: 2; *p* = 0.004) and non-Cochrane reviews (mean AMSTAR 2 score difference: 6; *p* = 0.003). Furthermore, SRs with declared conflicts of interests were of significantly lower methodological quality than SRs with no conflicts of interests (mean AMSTAR 2 score difference: 2; *p* = 0.004). None of the other characteristics had an impact on the methodological quality (see Table 5 for bivariate analyses).

**Table 5:** AMSTAR 2 scores according to characteristics of vaccination-related systematic reviews

Characteristics of systematic reviews	Yes^a^	No^a^	*p*-value^b^
Publication year after 2017^c^	11 (3–16)	9 (2–16)	0.004
Number of authors involved in systematic review ≥ 5^d^	10 (3–16)	9 (2–15)	0.28
Cochrane review	16 (15–16)	10 (2–15)	0.003
Type of included studies			
RCTs included	10 (5–16)	9 (4–15)	0.24
NRSIs included	10 (4–15)	10 (5–16)	0.46
Single-arm studies included	11 (8–15)	10 (4–16)	0.08
Funding by pharmaceutical company	8 (3–15)	10 (2–16)	0.05
Conflicts of interest	8 (3–15)	10 (2–16)	0.004

*NRSIs* Non-randomized studies of interventions, *RCTs* Randomized-controlled trials

^a^Median (range)

^b^Mann-Whitney U-test

^c^AMSTAR 2 was published first in 2017 [2]

^d^Median of included authors in review

**Fig. 3:**
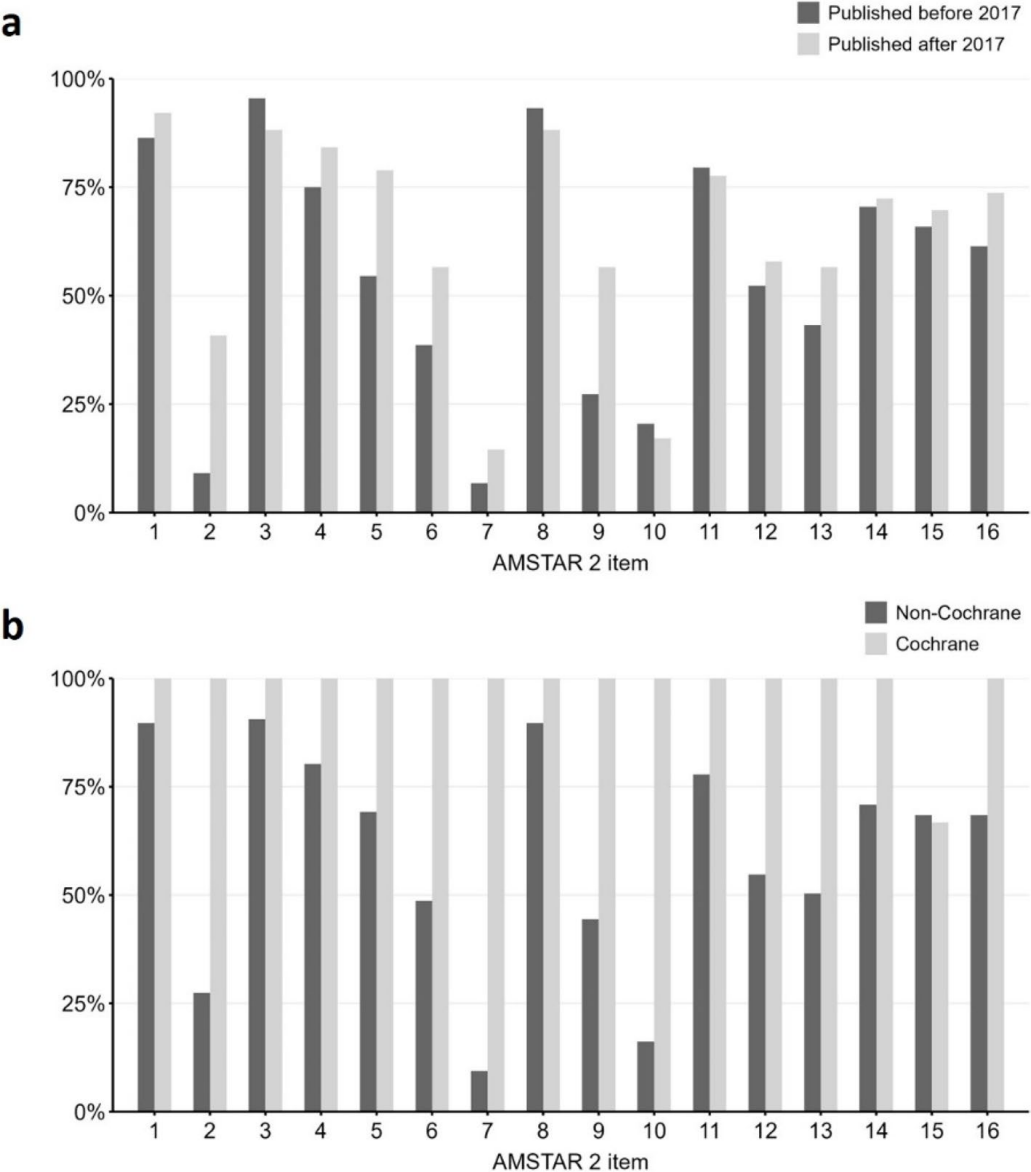
AMSTAR 2 scores at item level (1-16) given as a percentage of systematic reviews receiving a “yes”, “partial yes”, or “no meta-analysis conducted” in **a** Reviews published before (*n* = 44) vs. after 2017 (*n* = 76). Groups are significantly different for critical items 2 and 9 and non-critical item 5 (*p* < 0.05; chi-squared test); and **b** Cochrane reviews (*n* = 3) vs. non-Cochrane reviews (*n* = 117). Groups are significantly different for critical items 2 and 7 and non-critical items 10 and 12 (*p* < 0.05; Fisher’s exact test). *Item 1* PICO, *Item 2* Protocol registration, *Item 3* Study design, *Item 4* Adequacy of literature search strategy, *Item 5* Study selection in duplicate, *Item 6* Study extraction in duplicate, *Item 7* Justification for excluding individual studies, *Item 8* Study characteristics, *Item 9* Risk of bias assessment, *Item 10* Funding sources of the included studies, *Item 11* Appropriateness of meta-analytical methods, *Item 12* Impact of the risk of bias assessment of individual studies, *Item 13* Risk of bias interpretation, *Item 14* Discussion of heterogeneity, *Item 15* Publication bias, *Item 16* Management of conflicts of interest. Further information on AMSTAR 2 items 1–16 can be found in Table 1

To further investigate which individual AMSTAR 2 items contributed to these differences, we compared SRs published before and after 2017, Cochrane reviews and non-Cochrane reviews, and SRs from authors with and without CoI. SRs published after 2017 had significantly higher methodological quality than SRs published before 2017 because of protocol registration (40.8% vs. 9.1%, critical item 2), study selection in duplicate (78.9% vs. 54.5%, non-critical item 5), and adequate RoB assessment (56.6% vs. 27.3%, critical item 9) (all *p* < 0.05, see Fig. 3a). Cochrane reviews had significantly higher methodological quality than non-Cochrane reviews because of protocol registration (100.0% vs. 27.4%, critical item 2), justification for excluding individual studies (100.0% vs. 9.4%, critical item 7), reporting of funding sources of studies (100.0% vs. 16.2%, non-critical item 10), and reporting the impact of RoB assessment of individual studies (100.0% vs. 54.7%, non-critical item 12) (all *p* < 0.05, see Fig. 3b). Interestingly, only a quarter of SRs with declared CoI described their approach for managing these conflicts (*n* = 9/43, 20.9%, non-critical item 16).

The multivariable linear regression analysis showed that 26% of the variability of the methodological quality of the SRs was explained by the eight factors in the model (R^2^ = 0.26; adjusted R^2^ = 0.17; see Table 6 for analysis). However, only the publication year after 2017 (yes/no) and Cochrane review status (yes/no) had a significant independent influence on AMSTAR 2 score.

**Table 6:** Multivariable linear regression analysis: AMSTAR 2 score according to characteristics of vaccination-related systematic reviews (R^2^ = 0.26; adjusted R^2^ = 0.19)

Characteristics in the model	Beta	*T*	*p*-value
Publication year after 2017^a^	−1.730	−2.749	0.008
Number of authors involved in systematic review ≥ 5^b^	−0.121	−0.213	0.832
Cochrane review	−5.690	−3.217	0.002
Type of included studies			
RCTs included	0.010	0.012	0.990
NRSIs included	−0.335	−0.469	0.641
Single-arm studies included	−0.424	−0.417	0.678
Funding by pharmaceutical company	0.862	1.034	0.305
Conflicts of interest	0.548	0.768	0.445

*RCTs* Randomized-controlled trials, *NRSIs* Non-randomized studies of interventions

^a^AMSTAR 2 was published first in 2017 [2]

^b^Median of included authors in review

## Discussion

This methodological study used the AMSTAR 2 tool to assess the methodological quality of vaccination-related SRs and to identify sources and determining factors of critically low quality. We found only a minority of SRs of high quality, particularly those published in the Cochrane Library. The predominance of critically low-quality SRs in our random sample of vaccination-related SRs is concerning, considering their relevance in guiding global and national vaccination policies. Recent overviews of SRs on COVID-19 or influenza vaccination showed similar results regarding the proportion of critically low-rated SRs [11–14].

Our results showed that the most often unmet critical items of AMSTAR 2 were related to the justification for excluding individual studies and the protocol registration (items 7 and 2), leading to a critically low rating. This observation was also made in overviews of SRs in other areas of medicine, such as non-operative treatments for proximal humerus fractures [15]. Justifying the exclusion of individual studies in a SR increases transparency and reproducibility and supports the understanding of the eligibility criteria of the review. Protocol registration not only informs researchers about ongoing SRs, thereby helping to avoid redundancy, but also increases transparency and minimizes the potential for bias [16].

A recent bibliometric analysis of vaccination-related SRs showed that the proportion of SRs with high quality increased from 9% in 2017 to 22% in 2018 [6], which coincides with the release of AMSTAR 2 and the Cochrane Collaboration Handbook for review authors [2]. This might indicate an increasing awareness in the research community regarding methodological standards for the conduct of SRs. Making protocol registration in platforms like PROSPERO mandatory by publishers might further enhance transparency and minimize reporting bias [17, 18]. Other strategies may be enforcement of compliance with reporting guidelines such as PRISMA, or incentives for rigorously conducted SRs with greater recognition and support through funding agencies and academic institutions [19, 20].

So far, two studies have assessed the methodological quality of SRs on influenza vaccination [21, 22]. Using the first version of the AMSTAR tool, Remschmidt et al. also found that the Cochrane review status (Cochrane review vs. non-Cochrane review) had an impact on the methodological quality of SRs on influenza vaccination [21]. However, in their sample of SRs, major flaws were observed with regard to study selection in duplicate, inclusion of grey literature, and justification for excluding individual studies [21]. These results differ from those of our study, where Cochrane reviews differed from non-Cochrane reviews regarding protocol registration, justification for excluding individual studies, funding sources of the included studies, and the impact of the RoB assessment of individual studies (item 2, 7, 10, and 12). Additionally, our results showed that SRs published after 2017 (i.e., after publication of AMSTAR 2) were of higher methodological quality. Differences in the quality between SRs published after and before 2017 were due to protocol registration, study selection in duplicate, and RoB assessment (item 2, 5, and 9). However, direct comparison between both studies is limited due to differences between the two versions of the AMSTAR tool. In contrast to another study on vaccination-related SRs published from 2016 to 2019 [22], we did not observe an impact of industry sponsorship on the methodological quality of SRs. However, similar to that study [22], the same proportion of industry-funded SRs (15%) was found. Even if funding by the pharmaceutical industry did not influence the methodological quality of vaccination-related SRs, the management of CoI (item 16) was poorly described and remains crucial for interpreting SRs and drawing reliable conclusions. Nevertheless, multivariable analysis showed no significant independent relation between CoI and AMSTAR 2 score. Cochrane review status or publication date could be accountable for the observed effect.

Given the high proportion of SRs on vaccination with critically low methodological quality in our sample, the question remains how to deal with these SRs in daily practice, in particular in situations when resources or time constraints limit the ability to conduct a new, more rigorous SR. The median AMSTAR 2 score of the included SRs in this study was 10, indicating that 62.5% of the 16 items were met. NITAGs, as recommendation-making bodies for immunization policies, can consider using these SRs despite their limitations. Available guidance can support NITAGs in the stepwise selection of SRs based on their methodological quality [23, 24]. Future research could explore the development of a guidance for the critical appraisal of SRs based on their individual AMSTAR 2 items in the decision-making process.

This study has two main limitations. First, due to limited resources, only a proportion of SRs selected by block-randomization could be analyzed. Since not all vaccination topics are represented in this sample (e.g., we do not have SRs on pneumococcal disease or rotavirus in the sample), the generalizability of our results might be questioned. Second, we acknowledge that calculating a summary score is not the recommended method for assessing methodological quality; however, this intuitive approach has already been adopted by others [22, 25] and we deemed it appropriate to use to facilitate descriptive analysis. Overall, this study provides an up-to-date overview on the methodological quality of vaccination-related SRs and provides a starting point for improving SR methodological standards.

## Conclusions

Our study found critical methodological shortcomings in vaccination-related SRs. By addressing limitations, authors of newly developed SRs can improve the quality and reliability of evidence synthesis, thereby supporting robust decision-making in vaccination policy and practice. SRs provide the evidence base for public health decisions; therefore, ensuring their high methodological quality is essential. Future efforts should prioritize adherence to established methodological standards to enhance the impact of SRs and to inform evidence-based decision-making.

## Supplementary Information

Additional file 1: Study protocol.

Additional file 2: Search strategy.

Additional file 3: Characteristics of included systematic reviews; *n* = 120. 

Additional file 4: Methodological quality assessment of included systematic reviews using AMSTAR 2; *n* = 120.

## Data Availability

The datasets supporting the conclusions of this article are included within the article and its additional files. A list of the SRs not considered in this analysis is available upon request.

## Abbreviations

AMSTAR 2: A Measurement Tool to Assess systematic Reviews 2
CoI: Conflicts of interest
HPV: Human papillomavirus
HIB: Haemophilus influenzae type b
NITAG: National Immunization Technical Advisory Group
NRSI: Non-randomized studies of interventions
RCT: Randomized-controlled trials
RoB: Risk of bias
ROBIS: Risk Of Bias In Systematic reviews
SR: Systematic review
TBE: Tick-borne encephalitis
WHO: World Health Organization

## References

[1] Whiting P, Savovic J, Higgins JP, Caldwell DM, Reeves BC, Shea B, et al. ROBIS: A new tool to assess risk of bias in systematic reviews was developed. J Clin Epidemiol. 2016;69:225–34. 10.1016/j.jclinepi.2015.06.005. 26092286 10.1016/j.jclinepi.2015.06.005 PMC4687950

[2] Shea BJ, Reeves BC, Wells G, Thuku M, Hamel C, Moran J, et al. AMSTAR 2: a critical appraisal tool for systematic reviews that include randomised or non-randomised studies of healthcare interventions, or both. BMJ. 2017;358:j4008. 10.1136/bmj.j4008. 28935701 10.1136/bmj.j4008 PMC5833365

[3] Shea BJ, Hamel C, Wells GA, Bouter LM, Kristjansson E, Grimshaw J, et al. AMSTAR is a reliable and valid measurement tool to assess the methodological quality of systematic reviews. J Clin Epidemiol. 2009;62(10):1013–20. 10.1016/j.jclinepi.2008.10.009. 19230606 10.1016/j.jclinepi.2008.10.009

[4] Pilic A, Henaff L, Steffen C, Wichmann O, Piechotta V, Harder T. How do national immunization technical advisory groups assess and use evidence: findings from the SYSVAC survey. Vaccine. 2025;43:126538. 10.1016/j.vaccine.2024.126538. 39571357 10.1016/j.vaccine.2024.126538

[5] Immunization Agenda 2030 Partners. Immunization agenda 2030: a global strategy to leave no one behind. Vaccine. 2024;42:S5–14. 10.1016/j.vaccine.2022.11.042. 39004466 10.1016/j.vaccine.2022.11.042

[6] Pilic A, Henaff L, Steffen CA, Linß HH, Dreyer AI, Batke M, et al. Research trends of vaccination-related systematic reviews, 2011–2023: a bibliometric analysis. Publications. 2025;13(2):25. 10.3390/publications13020025.

[7] Adjagba A, Henaff L, Duclos P. The NITAG resource centre (NRC): one-stop shop towards a collaborative platform. Vaccine. 2015;33(36):4365–7. 10.1016/j.vaccine.2015.06.106. 26165917 10.1016/j.vaccine.2015.06.106

[8] SYSVAC. https://www.nitag-resource.org/sysvac-systematic-reviews. Accessed 18 Dec 2024.

[9] Epistemonikos. Living Overview of Evidence repository https://app.iloveevidence.com/loves/5e6fdb9669c00e4ac072701d. Accessed 01 June 2024.

[10] Jo CL, Burchett H, Bastías M, Campbell P, Gamage D, Henaff L, et al. Using existing systematic reviews for developing vaccination recommendations: Results of an international expert workshop. Vaccine. 2021;39(23):3103–10. 10.1016/j.vaccine.2021.04.045. PubMed PMID: WOS:000652613400001. 10.1016/j.vaccine.2021.04.045 33965256

[11] Yang D, Tian J, Shen C, Li Q. An overview and single-arm meta-analysis of immune-mediated adverse events following COVID-19 vaccination. Front Pharmacol. 2024;15. 10.3389/fphar.2024.1308768. PubMed PMID: 2030346077. 10.3389/fphar.2024.1308768 PMC11200080 38933672

[12] Angeletti PM, Marchi S, Trombetta CM, Altobelli E. Flu vaccine administration in the period before SARS-CoV-2 infection and its outcomes: an umbrella review. Prev Med Rep. 2024;38. 10.1016/j.pmedr.2023.102575. PubMed PMID: 2029625204. 10.1016/j.pmedr.2023.102575 PMC10820254 38283956

[13] Okoli GN, Reddy VK, Lam OLT, Racovitan F, Al-Yousif Y, Askin N. Characteristics and methodological standards across systematic reviews with meta-analysis of efficacy and/or effectiveness of influenza vaccines: an overview of reviews. Infect Dis. 2022;54(12):861–80. 10.1080/23744235.2022.2114537. PubMed PMID: 2018753704. 10.1080/23744235.2022.2114537 36000220

[14] Carregaro RL, Roscani ANCP, Raimundo ACS, Ferreira L, Vanni T, da Graca Salomao M, et al. Immunogenicity and safety of inactivated quadrivalent influenza vaccine compared with the trivalent vaccine for influenza infection: an overview of systematic reviews. BMC Infect Dis. 2023;23(1). 10.1186/s12879-023-08541-0. PubMed PMID: 2025175980. 10.1186/s12879-023-08541-0 PMC10463610 37644401

[15] Sandau N, Buxbom P, Hróbjartsson A, Harris IA, Brorson S. The methodological quality was low and conclusions discordant for meta-analyses comparing proximal humerus fracture treatments: a meta-epidemiological study. J Clin Epidemiol. 2022;142:100–9. 10.1016/j.jclinepi.2021.10.014. 34718123 10.1016/j.jclinepi.2021.10.014

[16] Higgins JPT, Thomas J, Chandler J, Cumpston M, Li T, Page MJ, et al. Cochrane handbook for systematic reviews of interventions version 6.5 (updated August 2024). The cochrane collaboration, 2024;2024. www.training.cochrane.org/handbook.

[17] Schiavo JH. PROSPERO: An international register of systematic review protocols. Med Ref Serv Q. 2019;38(2):171–80. 10.1080/02763869.2019.1588072. 31173570 10.1080/02763869.2019.1588072

[18] Higgins JPT, Thomas J, Chandler J, Cumpston M, Li T, Page MJ, et al. Cochrane handbook for systematic reviews of interventions version 5.2.0 (updated June 2017). The cochrane collaboration, 2017;2017. www.handbook.cochrane.org.

[19] Tugwell P, Tovey D. PRISMA 2020. J Clin Epidemiol. 2021;134:A5–6. 10.1016/j.jclinepi.2021.04.008. 34016443 10.1016/j.jclinepi.2021.04.008

[20] Glasziou P. The role of open access in reducing waste in medical research. PLoS Med. 2014;11(5):e1001651. 10.1371/journal.pmed.1001651. 24866475 10.1371/journal.pmed.1001651 PMC4035270

[21] Remschmidt C, Wichmann O, Harder T. Methodological quality of systematic reviews on influenza vaccination. Vaccine. 2014;32(15):1678–84. 10.1016/j.vaccine.2014.01.060. PubMed PMID: 53019559. 24513008 10.1016/j.vaccine.2014.01.060

[22] Pieper D, Hellbrecht I, Zhao L, Baur C, Pick G, Schneider S, et al. Impact of industry sponsorship on the quality of systematic reviews of vaccines: a cross-sectional analysis of studies published from 2016 to 2019. Syst Rev. 2022;11(1):174. 10.1186/s13643-022-02051-x. 35996186 10.1186/s13643-022-02051-x PMC9395849

[23] Pilic A, Reda S, Jo CL, Burchett H, Bastías M, Campbell P, et al. Use of existing systematic reviews for the development of evidence-based vaccination recommendations: guidance from the SYSVAC expert panel. Vaccine. 2023;41(12):1968–78. 10.1016/j.vaccine.2023.02.027. 36804216 10.1016/j.vaccine.2023.02.027 PMC10015272

[24] Lorenz RC, Jenny M, Jacobs A, Matthias K. Fast-and-frugal decision tree for the rapid critical appraisal of systematic reviews. Res Synth Methods. 2024;15(6):1049–59. 10.1002/jrsm.1754. 39234960 10.1002/jrsm.1754

[25] Pieper D, Lorenz RC, Rombey T, Jacobs A, Rissling O, Freitag S, et al. Authors should clearly report how they derived the overall rating when applying AMSTAR 2—a cross-sectional study. J Clin Epidemiol. 2021;129:97–103. 10.1016/j.jclinepi.2020.09.046. 33049325 10.1016/j.jclinepi.2020.09.046

