# Supplementary material for: Methodological quality of systematic intervention reviews on vaccination
Source: Syst Rev. 2026 Jan 7;15:43. doi: 10.1186/s13643-025-03052-2 (PMC12870254; doi:10.1186/s13643-025-03052-2)
Supplement: Supplementary file 1 — Additional file 1: Study protocol. [file 13643_2025_3052_MOESM1_ESM.docx]

Additional file 1: study protocol

**Protocol**

Methodological quality of systematic intervention reviews on vaccination, published from 2011 to 2023

*First version finalized on 21 August 2024*

## OBJECTIVES AND RESEARCH QUESTIONS

**Objectives**

1. Identify and assess methodological quality of systematic reviews on the efficacy or effectiveness of vaccines using the AMSTAR 2 tool
2. Investigate which characteristics (items) influence the quality of these reviews

**Research questions**

1. What characteristics of systematic reviews are associated with high methodological quality?
2. How does the methodological quality of Cochrane reviews compare to non-Cochrane reviews, and which key elements differ most?

## METHODOLOGY

**Data source**

- AMSTAR 2 assessed systematic reviews included in SYSVAC registry, systematically incorporating systematic reviews on immunization from MEDLINE/PubMed, Embase, Cochrane Library and the Living Overview of Evidence Platform

**Eligibility criteria**

- AMSTAR 2 assessed systematic reviews on the efficacy/effectiveness of vaccines published 2011-2023, and written in English language
- Exclusion of reviews focused on other topics than efficacy/effectiveness, and umbrella reviews due to inapplicability of AMSTAR 2 tool

**Data Extraction**

- Two independent reviewers will extract systematic review characteristics (see below). Disagreements will be resolved by consensus.

Systematic review characteristics:

- Year of publication (2011-2023)
- Number of authors involved in systematic review
- Background of corresponding author (academia, industry, etc.)
- Country of corresponding author
- Cochrane review (yes/no)
- Type of included studies: (yes/no/not reported)
  - RCTs included
  - NRSIs included
  - Single-arm studies included
- Funding by pharmaceutical company (yes/no/not reported)
- Conflict of interests (yes/no/not reported)
- AMSTAR 2 summary score (0-16)

**Quality Assessment**

- A random sample of 120 AMSTAR 2 assessed systematic reviews on HPV, influenza or other diseases/pathogens with <50 systematic reviews included in the SYSVAC registry will be selected (40 systematic reviews each)
- All 16 AMSTAR 2 items will be analyzed in terms of "yes", “partial yes”, "no", or "no meta-analysis conducted” responses, and an AMSTAR 2 summary score will be calculated by summing the "yes" and “partial yes” responses.

Adjustments against the original AMSTAR 2 tool:

- Item 2: Did the report of the review contain an explicit statement that the review methods were established prior to the conduct of the review and did the report justify any significant deviations from the protocol?
  - No Partial Yes option as generally binary outcome
- Item 3: Did the review authors explain their selection of the study designs for inclusion in the review?
  - This item receives a Yes whenever any information on study type is provided, regardless of the existence of an explanation, e.g. “we included RCTs, cohort studies and case-control studies”
- Item 4: Did the review authors use a comprehensive literature search strategy? Subitem: justified publication restrictions (e.g. language).
  - This subitem receives a Yes whenever any information on restrictions is provided, regardless of the existence of an explanation, e.g. “we included studies in English and French”; this subitem receives a No when no restriction information at all is provided
- Item 7: Did the review authors provide a list of excluded studies and justify the exclusions?
  - No Partial Yes option as generally binary outcome
- Item 8: Did the review authors describe the included studies in adequate detail?
  - No Partial Yes option as generally binary outcome
- Item 9: Did the review authors use a satisfactory technique for assessing the risk of bias (RoB) in individual studies that were included in the review?
  - No Partial Yes option as generally binary outcome
- Item 11, 12 and 15: Mention of the word “meta-analysis” is a must to consider assessment of this item
- Item 15: If they performed quantitative synthesis did the review authors carry out an adequate investigation of publication bias (small study bias) and discuss its likely impact on the results of the review?
  - This item receives a Yes whenever “publication bias” term mentioned as such and/or Begg’s or Egger’s test performed, incl. graphical plot or if there is an explanation why no publication bias was investigated (e.g. due to low numbers of studies)

**Data analysis plan**

- Descriptive analysis: Results will be displayed as median and range or n (%), as appropriate. (table)
- Comparative analysis
- Single 16 AMSTAR 2 items between Cochrane reviews and non-Cochrane reviews using Chi-squared test (bar chart)
- AMSTAR 2 summary scores according to systematic review characteristics using Mann-Whitney U-test (yes/no) (table):
  - - Publication year after 2017 (AMSTAR 2 was published first in 2017)
    - Number of authors involved in systematic review (median of number of authors; yes/no)
    - Cochrane review (yes/no)
    - Type of included studies (yes/no)
      - RCTs included
      - NRSIs included
      - Single-arm studies included
    - Funding by pharmaceutical company (yes/no)
    - Conflicts of interest (yes/no)
- Multivariable linear regression analysis: influence of systematic review characteristics on AMSTAR 2 summary scores (table):
  - Publication year after 2017 (AMSTAR 2 was published first in 2017)
  - Number of authors involved in systematic review (median of number of authors; yes/no)
  - Cochrane review (yes/no)
  - Type of included studies (yes/no)
    - RCTs included
    - NRSIs included
    - Single-arm studies included
  - Funding by pharmaceutical company (yes/no)
  - Conflicts of interest (yes/no)
- Statistical approach: Data analysis will be done using R. A p-value of less than 0.05 considered statistically significant.
- Data Visualization: tables or bar charts

**Protocol amendments**

- Comparative analysis of single 16 AMSTAR 2 items of further review characteristics that showed significant differences in bivariate analysis (05 December 2024)
- Subgroup analysis of critical items of critically low-rated systematic reviews (05 December 2024)
