# Supplementary material for: Methodological quality of systematic intervention reviews on vaccination
Source: Syst Rev. 2026 Jan 7;15:43. doi: 10.1186/s13643-025-03052-2 (PMC12870254; doi:10.1186/s13643-025-03052-2)
Supplement: Supplementary file 2 — Additional file 2: Search strategy. [file 13643_2025_3052_MOESM2_ESM.docx]

Additional file 2: search strategy

### DATABASE SEARCH STRATEGY

**MEDLINE** via OVID 1946 to June 01, 2024

|  | Searches |
| --- | --- |
| 1 | (review or review, tutorial or review, academic).pt. |
| 2 | (medline or medlars or embase or pubmed or cochrane).tw,sh. |
| 3 | (scisearch or psychinfo or psycinfo).tw,sh. |
| 4 | (psychlit or psyclit).tw,sh. |
| 5 | cinahl.tw,sh. |
| 6 | ((hand adj2 search$) or (manual$ adj2 search$)).tw,sh. |
| 7 | (electronic database$ or bibliographic database$ or computeri?ed database$ or online database$).tw,sh. |
| 8 | (pooling or pooled or mantel haenszel).tw,sh. |
| 9 | (peto or dersimonian or der simonian or fixed effect).tw,sh. |
| 10 | (retraction of publication or retracted publication).pt. |
| 11 | or/2-10 |
| 12 | 1 and 11 |
| 13 | meta-analysis.pt. |
| 14 | meta-analysis.sh. |
| 15 | (meta-analys$ or meta analys$ or metaanalys$).tw,sh. |
| 16 | (systematic$ adj5 review$).tw,sh. |
| 17 | (systematic$ adj5 overview$).tw,sh. |
| 18 | (quantitativ$ adj5 review$).tw,sh. |
| 19 | (quantitativ$ adj5 overview$).tw,sh. |
| 20 | (quantitativ$ adj5 synthesis$).tw,sh. |
| 21 | (methodologic$ adj5 review$).tw,sh. |
| 22 | (methodologic$ adj5 overview$).tw,sh. |
| 23 | (integrative research review$ or research integration).tw. |
| 24 | or/13-23 |
| 25 | 12 or 24 |
| 26 | exp immunization/ |
| 27 | exp Immunization Programs/ |
| 28 | exp vaccines/ |
| 29 | (immunisation or immunization or immunise or immunize or immunising or immunizing or immunised or immunized or immunises or immunizes or (vaccine and immunity)).ti,ab. |
| 30 | (vaccine$ or vaccination$ or vaccinate$ or vaccinating).ti,ab. |
| 31 | or/26-30 |
| 32 | 25 and 31 |
| 33 | limit 32 to humans |
| 34 | (201101* or 201102* … or 202312*).ed. |
| 35 | 33 and 34 |

* 34 needs to be changed according to the dates required for the update

**EMBASE** to June 01, 2024

|  | Searches |
| --- | --- |
| 1 | exp immunization/ |
| 2 | exp vaccine/ |
| 3 | (immunisation or immunization or immunise or immunize or immunising or immunizing or immunised or immunized or immunises or immunizes or (vaccine and immunity)).ti,ab. |
| 4 | (vaccine$ or vaccination$ or vaccinate$ or vaccinating).ti,ab. |
| 5 | or/1-4 |
| 6 | exp review/ |
| 7 | (literature adj3 review$).ti,ab . |
| 8 | exp meta analysis/ |
| 9 | exp systematic review/ |
| 10 | or/6-9 |
| 11 | (medline or medlars or embase or pubmed or cinahl or amed or psychlit or psyclit or psychinfo or psycinfo or scisearch or cochrane).ti,ab. |
| 12 | (retraction of publication or retracted publication).pt. |
| 13 | 11 or 12 |
| 14 | 10 and 13 |
| 15 | (systematic adj2 (review$ or overview)).ti,ab. |
| 16 | ((meta$anal$ or meta) and anal$ or meta anal$ or metaanal$ or metanal$).ti,ab. |
| 17 | 14 or 15 or 16 |
| 18 | 5 and 17 |
| 19 | Limit 18 to humans |
| 20 | (201101* or … 202312*).ed |
| 21 | 19 and 20 |

**The Cochrane Library of Systematic Reviews** to June 01, 2024

|  | Searches |
| --- | --- |
| 1 | MeSH descriptor: [Vaccines] explode all trees |
| 2 | MeSH descriptor: [Immunization] explode all trees |
| 3 | MeSH descriptor: [Immunization Programs] explode all trees |
| 4 | ((immunisation or immunization or immunise or immunize or immunising or immunizing or immunised or immunized or immunises or immunizes or (vaccine and immunity))):ti,ab,kw |
| 5 | (vaccine$ or vaccination$ or vaccinate$ or vaccinating or (mass and campaign)):ti,ab,kw |
| 6 | #1 OR #2 OR #3 OR #4 OR #5 |

**The Living Overview of Evidence** repository to June 01, 2024: All Systematic reviews to be found in the repository under category *Vaccines [1]*.

### REFERENCES

1. Epistemonikos. Living Overview of Evidence repository <https://app.iloveevidence.com/loves/5e6fdb9669c00e4ac072701d>. Accessed 01 June 2024.
