# Supplementary material for: Methodological quality of systematic intervention reviews on vaccination
Source: Syst Rev. 2026 Jan 7;15:43. doi: 10.1186/s13643-025-03052-2 (PMC12870254; doi:10.1186/s13643-025-03052-2)
Supplement: Supplementary file 4 — Additional file 4: Methodological quality assessment of included systematic reviews using AMSTAR 2; n = 120. [file 13643_2025_3052_MOESM4_ESM.docx]

Additional file 4: Methodological quality assessment of included systematic reviews using AMSTAR 2; n=120

| **First author, year** | **I1** | **I2** | **I3** | **I4** | **I5** | **I6** | **I7** | **I8** | **I9** | **I10** | **I11** | **I12** | **I13** | **I14** | **I15** | **I16** | **Rating** | **Score** |
| --- | --- | --- | --- | --- | --- | --- | --- | --- | --- | --- | --- | --- | --- | --- | --- | --- | --- | --- |
| Aaby et al., 2022 [1] | N | N | Y | PY | Y | N | N | Y | N | N | N | N | Y | N | Y | Y | critically low | 7 |
| Akter et al., 2023 [2] | Y | N | Y | PY | Y | Y | Y | Y | N | N | NM | NM | Y | Y | NM | Y | critically low | 13 |
| Aumatell et al., 2011 [3] | Y | N | Y | N | N | N | N | Y | N | N | NM | NM | Y | Y | NM | N | critically low | 8 |
| Bartoszko et al., 2018 [4] | Y | Y | Y | PY | Y | Y | N | Y | Y | N | Y | Y | Y | Y | N | N | critically low | 12 |
| Belongia et al., 2016 [5] | Y | N | Y | PY | Y | N | N | Y | N | N | Y | N | N | Y | Y | N | critically low | 8 |
| Belongia et al., 2017 [6] | Y | N | Y | PY | Y | N | N | Y | N | N | N | N | N | Y | N | N | critically low | 6 |
| Bergman et al., 2019 [7] | Y | Y | Y | PY | Y | Y | Y | Y | Y | Y | Y | Y | Y | Y | Y | Y | high | 16 |
| Beyer et al., 2020 [8] | Y | N | Y | PY | N | Y | N | N | N | N | N | N | N | N | N | Y | critically low | 5 |
| Boddington et al., 2021 [9] | Y | Y | Y | PY | Y | Y | N | Y | Y | N | Y | N | Y | Y | N | Y | critically low | 12 |
| Bohn-Goldbaum et al., 2022 [10] | Y | Y | Y | PY | N | N | N | Y | Y | N | Y | N | Y | Y | N | Y | critically low | 10 |
| Breteler et al., 2013 [11] | Y | N | Y | PY | Y | N | N | Y | Y | N | Y | N | Y | Y | N | Y | critically low | 10 |
| Camilloni et al., 2015 [12] | Y | N | Y | N | N | N | N | Y | N | N | NM | NM | N | Y | NM | N | critically low | 7 |
| Casanova et al., 2016 [13] | Y | N | Y | PY | Y | Y | N | Y | N | N | NM | NM | Y | N | NM | Y | critically low | 11 |
| Caspard et al., 2016 [14] | Y | N | Y | PY | N | N | N | Y | N | N | NM | NM | N | Y | NM | N | critically low | 8 |
| Caspard et al., 2017 [15] | Y | N | Y | N | Y | N | N | Y | N | N | N | N | N | N | N | N | critically low | 4 |
| Chan et al., 2014 [16] | Y | N | Y | N | N | Y | N | Y | Y | N | Y | Y | Y | Y | Y | Y | critically low | 11 |
| Choi et al., 2022 [17] | Y | Y | Y | PY | Y | Y | N | Y | Y | N | Y | N | N | Y | N | Y | critically low | 11 |
| Clar et al., 2015 [18] | Y | Y | Y | PY | Y | Y | Y | Y | Y | Y | Y | Y | Y | Y | N | Y | low | 15 |
| Coelho et al., 2015 [19] | Y | N | Y | PY | Y | N | N | Y | N | N | N | N | N | Y | N | Y | critically low | 7 |
| Coleman et al., 2021 [20] | Y | Y | Y | PY | Y | Y | N | Y | N | N | Y | N | Y | Y | Y | N | critically low | 11 |
| Conti et al., 2023 [21] | Y | N | Y | N | Y | N | N | Y | Y | N | N | N | Y | Y | N | Y | critically low | 8 |
| Corder et al., 2020 [22] | N | N | Y | N | N | N | N | Y | N | N | NM | NM | N | N | NM | Y | critically low | 6 |
| Couto et al., 2014 [23] | Y | N | Y | PY | Y | Y | N | Y | N | Y | N | Y | Y | N | N | Y | critically low | 10 |
| Cuningham et al., 2019 [24] | Y | N | Y | PY | Y | N | N | Y | Y | N | N | Y | Y | Y | Y | Y | critically low | 11 |
| da Costa et al., 2014 [25] | Y | Y | Y | PY | Y | Y | N | Y | N | N | Y | N | Y | Y | Y | Y | critically low | 12 |
| da Silveira et al., 2019 [26] | Y | N | Y | PY | Y | N | Y | Y | Y | Y | Y | N | N | Y | N | Y | critically low | 11 |
| D'Addario et al., 2017 [27] | Y | N | Y | N | Y | N | Y | Y | Y | N | Y | N | N | Y | N | N | critically low | 8 |
| Darvishian et al., 2014a [28] | Y | N | Y | PY | N | Y | N | Y | N | N | Y | N | N | Y | N | Y | critically low | 8 |
| Darvishian et al., 2014b [29] | Y | N | Y | N | N | Y | N | Y | N | N | Y | N | N | Y | N | Y | critically low | 7 |
| de Lejarazu-Leonardo et al., 2021 [30] | N | N | N | N | N | N | N | N | N | N | NM | NM | N | N | NM | N | critically low | 3 |
| Delere et al., 2014 [31] | Y | Y | Y | PY | N | N | N | Y | N | Y | Y | Y | Y | Y | Y | N | critically low | 11 |
| Di Donato et al., 2021 [32] | Y | N | Y | PY | Y | N | N | Y | N | N | Y | N | Y | Y | N | Y | critically low | 9 |
| Diallo et al., 2021 [33] | Y | Y | Y | N | Y | N | N | Y | Y | Y | Y | Y | Y | Y | Y | Y | critically low | 13 |
| DiazGranados et al., 2012 [34] | Y | N | Y | PY | N | N | N | Y | N | N | N | N | N | Y | Y | N | critically low | 6 |
| Domnich et al., 2017 [35] | Y | N | Y | PY | Y | Y | N | Y | N | N | Y | N | N | Y | Y | Y | critically low | 10 |
| Domnich et al., 2022 [36] | Y | Y | Y | PY | Y | Y | Y | Y | Y | Y | Y | N | Y | Y | Y | Y | high | 15 |
| Doyon-Plourde et al., 2019 [37] | Y | Y | Y | PY | Y | Y | N | Y | Y | N | NM | NM | Y | Y | NM | Y | low | 14 |
| Dzanibe et al., 2018 [38] | Y | N | N | PY | N | N | N | Y | N | N | NM | NM | N | N | NM | N | critically low | 6 |
| Ellingson et al., 2023 [39] | Y | N | Y | PY | Y | Y | N | Y | Y | Y | NM | NM | Y | N | NM | N | critically low | 12 |
| Eriksen et al., 2022 [40] | Y | Y | Y | PY | Y | N | N | Y | Y | N | N | Y | Y | Y | Y | N | critically low | 11 |
| Faust et al., 2019 [41] | Y | N | Y | PY | N | N | N | N | Y | N | NM | NM | N | Y | NM | Y | critically low | 9 |
| Feng et al., 2018 [42] | Y | N | Y | PY | Y | N | N | Y | N | N | Y | N | N | N | Y | N | critically low | 7 |
| Friedman et al., 2019 [43] | Y | Y | Y | PY | Y | Y | N | Y | N | N | NM | NM | N | N | NM | N | critically low | 10 |
| Fukuta et al., 2019 [44] | Y | N | Y | PY | N | Y | N | Y | Y | N | Y | N | N | N | N | N | critically low | 7 |
| Garland et al., 2016 [45] | N | N | Y | PY | Y | N | N | Y | N | N | NM | NM | N | N | NM | Y | critically low | 8 |
| Gartner et al., 2022 [46] | Y | N | Y | PY | N | N | N | Y | Y | N | NM | NM | Y | N | NM | Y | critically low | 10 |
| Genovese et al., 2018 [47] | Y | N | Y | PY | Y | Y | N | Y | N | N | N | N | N | Y | Y | Y | critically low | 9 |
| Goodman et al., 2022 [48] | Y | Y | Y | PY | N | N | N | N | N | N | NM | NM | Y | Y | NM | N | critically low | 9 |
| Gotuzzo et al., 2013 [49] | N | N | Y | PY | Y | Y | N | Y | N | N | NM | NM | Y | N | NM | N | critically low | 9 |
| Grassly, 2014 [50] | Y | N | N | N | N | N | N | N | N | N | N | N | N | N | N | Y | critically low | 2 |
| Griffiths et al., 2012 [51] | Y | N | Y | N | Y | Y | N | Y | Y | N | N | N | Y | Y | Y | Y | critically low | 10 |
| Guo et al., 2023 [52] | Y | N | Y | N | Y | Y | N | Y | Y | Y | Y | N | Y | Y | N | Y | critically low | 11 |
| Hamad et al., 2021 [53] | Y | N | Y | PY | Y | Y | N | Y | Y | N | N | Y | Y | Y | Y | Y | critically low | 12 |
| Harder et al., 2018 [54] | Y | Y | Y | PY | Y | Y | Y | Y | Y | Y | NM | NM | Y | N | NM | Y | high | 15 |
| Hawken et al., 2012 [55] | N | N | Y | N | N | N | N | Y | N | N | NM | NM | N | N | NM | N | critically low | 5 |
| Infante et al., 2020 [56] | Y | Y | Y | N | Y | Y | N | Y | N | N | N | N | N | Y | N | Y | critically low | 8 |
| Jackson et al., 2013 [57] | Y | N | Y | PY | N | N | N | Y | N | N | Y | Y | Y | Y | N | Y | critically low | 9 |
| Jones-Gray et al., 2023 [58] | Y | Y | Y | PY | Y | Y | N | Y | Y | N | Y | Y | N | Y | Y | N | critically low | 12 |
| Kechagias et al., 2022 [59] | Y | Y | Y | PY | Y | Y | N | Y | Y | N | Y | Y | Y | Y | Y | Y | low | 14 |
| Kling et al., 2022 [60] | Y | Y | Y | PY | Y | N | Y | Y | Y | N | N | N | Y | Y | N | Y | critically low | 11 |
| Kuo et al., 2012 [61] | Y | N | Y | PY | N | N | N | Y | N | N | NM | NM | N | Y | N | Y | critically low | 9 |
| Lansbury et al., 2017 [62] | Y | N | Y | Y | Y | Y | N | Y | Y | N | Y | Y | Y | Y | Y | Y | critically low | 13 |
| LeBras et al., 2017 [63] | Y | N | Y | PY | Y | Y | N | Y | N | N | NM | N | N | N | NM | Y | critically low | 10 |
| Lee et al., 2017 [64] | Y | N | Y | N | Y | N | N | Y | Y | N | Y | Y | Y | Y | Y | Y | critically low | 11 |
| Lee et al., 2018 [65] | Y | N | Y | N | N | N | N | Y | Y | N | N | N | N | Y | N | Y | critically low | 6 |
| Lee et al., 2021 [66] | Y | N | Y | PY | Y | Y | N | Y | N | N | N | N | N | Y | Y | N | critically low | 8 |
| Lei et al., 2017 [67] | Y | N | Y | Y | Y | N | N | Y | Y | N | N | N | N | Y | Y | Y | critically low | 9 |
| Leung et al., 2016 [68] | Y | N | Y | PY | N | N | N | Y | N | N | NM | NM | N | N | NM | N | critically low | 7 |
| Leung et al., 2017 [69] | N | N | N | N | N | N | N | Y | N | N | NM | NM | N | N | NM | Y | critically low | 5 |
| Li et al., 2021 [70] | Y | Y | Y | PY | Y | Y | N | Y | Y | N | N | N | Y | Y | Y | Y | critically low | 12 |
| Lichter et al., 2020 [71] | Y | Y | Y | PY | Y | Y | N | Y | Y | Y | Y | Y | Y | Y | N | Y | critically low | 13 |
| Lu et al., 2011 [72] | Y | N | Y | PY | N | Y | N | Y | N | Y | Y | N | N | Y | Y | N | critically low | 9 |
| Lukacs et al., 2020 [73] | Y | Y | Y | PY | Y | Y | N | Y | Y | N | Y | Y | Y | Y | Y | Y | low | 14 |
| Mac Eochagain et al., 2022 [74] | Y | Y | Y | PY | Y | Y | N | Y | Y | N | Y | Y | N | Y | Y | Y | critically low | 13 |
| Malagón et al., 2012 [75] | Y | N | Y | PY | Y | Y | N | Y | N | Y | Y | N | N | Y | N | N | critically low | 9 |
| Malisheni et al., 2017 [76] | Y | N | Y | PY | N | N | N | Y | Y | N | Y | N | Y | Y | Y | Y | critically low | 10 |
| Markowitz et al., 2018 [77] | Y | N | Y | PY | Y | Y | N | Y | N | N | NM | NM | N | Y | NM | Y | critically low | 11 |
| Markowitz et al., 2022 [78] | Y | N | Y | PY | Y | Y | N | Y | Y | N | NM | NM | Y | N | NM | Y | critically low | 12 |
| Mavundza et al., 2020 [79] | Y | Y | Y | PY | Y | Y | Y | Y | Y | N | NM | NM | Y | Y | NM | Y | high | 15 |
| McGirr et al., 2019 [80] | Y | N | Y | PY | Y | Y | N | Y | Y | N | N | N | N | Y | N | N | critically low | 8 |
| Miazga et al., 2023 [81] | Y | N | Y | PY | Y | N | Y | Y | Y | N | NM | NM | N | Y | NM | Y | critically low | 12 |
| Montagnani et al., 2014 [82] | N | N | Y | PY | N | N | N | N | N | N | NM | NM | N | N | NM | Y | critically low | 6 |
| Mousavi et al., 2016 [83] | Y | N | Y | PY | Y | N | N | Y | N | N | Y | N | Y | Y | Y | Y | critically low | 10 |
| Nielsen et al., 2021 [84] | Y | N | N | PY | N | N | N | Y | Y | N | NM | NM | N | N | NM | Y | critically low | 8 |
| Ott et al., 2012 [85] | Y | N | Y | PY | N | N | N | Y | N | N | NM | NM | Y | N | NM | Y | critically low | 9 |
| Pan et al., 2022 [86] | Y | N | N | PY | N | N | N | Y | N | N | Y | N | N | Y | Y | Y | critically low | 7 |
| Pittet et al., 2020 [87] | Y | N | Y | PY | N | N | N | Y | N | N | NM | NM | N | N | NM | Y | critically low | 8 |
| Pittet et al., 2022 [88] | Y | N | Y | PY | Y | N | N | Y | Y | N | Y | Y | Y | Y | Y | Y | critically low | 12 |
| Ponduri et al., 2023 [89] | Y | Y | Y | PY | Y | Y | N | Y | N | N | Y | N | Y | Y | N | Y | critically low | 11 |
| Prandi et al., 2021 [90] | Y | N | Y | PY | Y | Y | N | N | N | N | NM | NM | N | N | NM | Y | critically low | 9 |
| Preiss et al., 2018 [91] | Y | N | N | PY | Y | N | Y | N | N | N | N | N | Y | Y | N | N | critically low | 6 |
| Puig-Barbera et al., 2022 [92] | Y | Y | Y | PY | Y | N | Y | Y | Y | Y | Y | Y | Y | Y | Y | Y | high | 15 |
| Rampa et al., 2020 [93] | Y | Y | N | PY | Y | N | N | Y | N | Y | NM | NM | N | N | NM | Y | critically low | 10 |
| Ren et al., 2022 [94] | Y | N | Y | PY | Y | N | N | Y | N | Y | Y | N | Y | Y | N | Y | critically low | 10 |
| Rey-Ares et al., 2012 [95] | Y | N | Y | PY | Y | N | N | Y | N | Y | Y | N | N | Y | N | Y | critically low | 9 |
| Rosado et al., 2023 [96] | Y | N | Y | PY | Y | Y | N | Y | N | Y | NM | NM | Y | Y | NM | Y | critically low | 13 |
| Sangar et al., 2015 [97] | Y | N | Y | PY | N | Y | N | Y | N | Y | N | N | N | N | N | N | critically low | 6 |
| Schwerdtle et al., 2018 [98] | Y | N | N | PY | Y | Y | N | N | N | N | Y | N | Y | Y | Y | Y | critically low | 9 |
| Silverii et al., 2023 [99] | Y | Y | Y | N | Y | Y | N | Y | Y | N | Y | N | Y | Y | Y | N | critically low | 11 |
| Staadegaard et al., 2022 [100] | Y | N | N | PY | Y | Y | N | Y | N | N | N | N | Y | Y | N | N | critically low | 7 |
| Steffen et al., 2021 [101] | N | N | Y | PY | N | N | N | N | N | N | NM | NM | Y | N | NM | N | critically low | 6 |
| Stuurman et al., 2017 [102] | N | N | Y | PY | Y | N | N | N | N | N | NM | NM | N | Y | NM | N | critically low | 7 |
| Tejada et al., 2017 [103] | Y | N | Y | PY | Y | Y | N | Y | Y | Y | Y | Y | Y | Y | Y | Y | critically low | 14 |
| Teo et al., 2017 [104] | Y | Y | Y | Y | Y | Y | Y | Y | Y | Y | Y | Y | Y | Y | Y | Y | high | 16 |
| Truelove et al., 2020 [105] | N | N | N | PY | Y | Y | N | N | N | N | N | N | N | N | N | Y | critically low | 4 |
| Ventura et al., 2022 [106] | Y | N | Y | N | Y | N | N | Y | Y | N | Y | Y | Y | N | Y | Y | critically low | 10 |
| Wang et al., 2014 [107] | Y | N | Y | PY | N | N | N | Y | Y | N | Y | Y | Y | Y | Y | N | critically low | 10 |
| Wang et al., 2021 [108] | Y | N | Y | N | Y | Y | N | Y | N | N | NM | NM | N | N | NM | N | critically low | 8 |
| Wang et al., 2022 [109] | Y | N | Y | N | Y | N | N | Y | N | N | NM | NM | N | Y | NM | N | critically low | 8 |
| Wei et al., 2023 [110] | Y | Y | Y | PY | Y | Y | N | Y | N | N | Y | N | N | Y | N | Y | critically low | 10 |
| Whitworth et al., 2020 [111] | Y | Y | Y | PY | Y | N | N | Y | N | N | NM | NM | Y | Y | NM | Y | critically low | 12 |
| Wilson et al., 2023 [112] | Y | N | Y | PY | Y | N | N | Y | Y | N | NM | NM | Y | N | NM | Y | critically low | 11 |
| Yeasmin et al., 2022 [113] | Y | Y | Y | PY | Y | N | N | Y | Y | N | NM | NM | N | Y | NM | Y | critically low | 12 |
| Yedlapati et al., 2021 [114] | Y | N | Y | PY | Y | Y | N | Y | Y | N | Y | N | N | Y | Y | N | critically low | 10 |
| Yin et al., 2018 [115] | Y | N | Y | PY | Y | Y | N | Y | N | N | Y | N | N | Y | Y | Y | critically low | 10 |
| Zeevaert et al., 2023 [116] | Y | Y | Y | Y | N | N | Y | Y | N | Y | Y | N | N | Y | N | Y | critically low | 10 |
| Zhang et al., 2020 [117] | Y | N | Y | N | N | Y | N | Y | Y | N | Y | Y | Y | Y | Y | Y | critically low | 11 |
| Zhang, J. et al., 2021 [118] | N | N | Y | PY | Y | Y | N | Y | Y | N | Y | Y | N | Y | Y | Y | critically low | 11 |
| Zhang, Z. et al., 2021 [119] | Y | N | Y | PY | Y | Y | N | Y | Y | N | Y | Y | Y | Y | Y | Y | critically low | 13 |
| Zizza et al., 2021 [120] | Y | Y | Y | PY | Y | Y | N | Y | Y | N | N | Y | Y | Y | N | Y | critically low | 12 |

AMSTAR 2: A Measurement Tool to Assess systematic Reviews 2; PY: Partial yes; I: Item; N: No; NM: No meta-analysis conducted; Y: Yes.

I1: PICO
I2: Protocol registration
I3: Study design
I4: Adequacy of literature search strategy
I5: Study selection in duplicate
I6: Data extraction in duplicate
I7: Justification for excluding individual studies
I8: Description of study characteristics
I9: Risk of bias assessment
I10: Funding of studies
I11: Appropriateness of meta-analytical methods
I12: Results of risk of bias assessment of individual studies
I13: Risk of bias interpretation
I14: Discussion of heterogeneity
I15: Publication bias
I16: Conflicts of interest
Further information on AMSTAR 2 items to be found in manuscripts’ **Table 1**.

REFERENCES

1. Aaby P, Nielsen S, Fisker AB, Pedersen LM, Welaga P, Hanifi SMA, et al. Stopping Oral Polio Vaccine (OPV) After Defeating Poliomyelitis in Low- and Middle-Income Countries: Harmful Unintended Consequences? Review of the Nonspecific Effects of OPV. Open Forum Infectious Diseases. 2022;9(8). doi: <https://doi.org/10.1093%2Fofid%2Fofac340>.

2. Akter F, Hasan TB, Alam F, Das A, Afrin S, Maisha S, et al. Effect of prior immunisation with smallpox vaccine for protection against human monkeypox: A systematic review. Reviews in Medical Virology. 2023. doi: <https://doi.org/10.1002/rmv.2444>.

3. Aumatell CM, Torrell JR, Zuckerman JN. Review of oral cholera vaccines: Efficacy in young children. Infection and Drug Resistance. 2011;4(1):155-60. doi: <http://dx.doi.org/10.2147/IDR.S10339>. PubMed PMID: 2012247367.

4. Bartoszko JJ, McNamara IF, Aras OAZ, Hylton DA, Zhang YB, Malhotra D, et al. Does consecutive influenza vaccination reduce protection against influenza: A systematic review and meta-analysis. Vaccine. 2018;1(24):3434-44. doi: <https://admin.abcsignup.com/files/F966A209-9637-40A1-B074-45E35B601661_37/23002/Sept2018_JC_Article_Oyer-Peterson.pdf>.

5. Belongia EA, Simpson MD, King JP, Sundaram ME, Kelley NS, Osterholm MT, et al. Variable influenza vaccine effectiveness by subtype: a systematic review and meta-analysis of test-negative design studies. The Lancet Infectious Diseases. 2016;16(8):942-51. doi: <http://dx.doi.org/10.1016/S1473-3099%2816%2900129-8>. PubMed PMID: 609669132.

6. Belongia EA, Skowronski DM, McLean HQ, Chambers C, Sundaram ME, De Serres G. Repeated annual influenza vaccination and vaccine effectiveness: review of evidence. Expert Review of Vaccines. 2017;16(7):723-36. doi: <http://dx.doi.org/10.1080/14760584.2017.1334554>. PubMed PMID: 616963294.

7. Bergman H, Buckley BS, Villanueva G, Petkovic J, Garritty C, Lutje V, et al. Comparison of different human papillomavirus (HPV) vaccine types and dose schedules for prevention of HPV-related disease in females and males. Cochrane Database Syst Rev. 2019;11(11):22. doi: <https://dx.doi.org/10.1002/14651858.CD013479>. PubMed PMID: 31755549.

8. Beyer WEP, Palache AM, Reperant LA, Boulfich M, Osterhaus A. Association between vaccine adjuvant effect and pre-seasonal immunity. Systematic review and meta-analysis of randomised immunogenicity trials comparing squalene-adjuvanted and aqueous inactivated influenza vaccines. Vaccine. 2020;38(7):1614-22. doi: <https://pure.eur.nl/ws/files/48291085/Repub_122918.pdf>. PubMed PMID: 31879122.

9. Boddington NL, Pearson I, Whitaker H, Mangtani P, Pebody RG. Effectiveness of Influenza Vaccination in Preventing Hospitalization Due to Influenza in Children: A Systematic Review and Meta-analysis. Clin Infect Dis. 2021;73(9):1722-32. doi: <https://researchonline.lshtm.ac.uk/id/eprint/4660543/1/SUBMISSION__Revision_Full%20Manuscript_Clean%20%281%29.pdf>. PubMed PMID: 33772586.

10. Bohn-Goldbaum E, Owen KB, Lee VYJ, Booy R, Edwards KM. Physical activity and acute exercise benefit influenza vaccination response: A systematic review with individual participant data meta-analysis. PLoS ONE. 2022;17(6):e0268625. doi: <https://dx.doi.org/10.1371/journal.pone.0268625>. PubMed PMID: 35704557.

11. Breteler JK, Tam JS, Jit M, Ket JC, De Boer MR. Efficacy and effectiveness of seasonal and pandemic A (H1N1) 2009 influenza vaccines in low and middle income countries: a systematic review and meta-analysis. Vaccine. 2013;31(45):5168-77. doi: <http://dx.doi.org/10.1016/j.vaccine.2013.08.056>. PubMed PMID: 24012574.

12. Camilloni B, Basileo M, Valente S, Nunzi E, Iorio AM. Immunogenicity of intramuscular MF59- Adjuvanted and intradermal administered influenza enhanced vaccines in subjects aged over 60: A literature review. Hum Vaccin Immunother. 2015;11(3):553-63. doi: <http://dx.doi.org/10.1080/21645515.2015.1011562>. PubMed PMID: 604117287.

13. Casanova L, Gobin N, Villani P, Verger P. Bias in the measure of the effectiveness of seasonal influenza vaccination among diabetics. Primary Care Diabetes. 2016;10(6):398-406. doi: <http://dx.doi.org/10.1016/j.pcd.2016.05.005>. PubMed PMID: 613646085.

14. Caspard H, Heikkinen T, Belshe RB, Ambrose CS. A systematic review of the efficacy of live attenuated influenza vaccine upon revaccination of children. Hum Vaccin Immunother. 2016;12(7):1721-7. doi: <http://dx.doi.org/10.1080/21645515.2015.1115164>. PubMed PMID: 611316549.

15. Caspard N, Mallory RM, Yu J, Ambrose CS. Live-Attenuated Influenza Vaccine Effectiveness in Children From 2009 to 2015-2016: A Systematic Review and Meta-Analysis. Open Forum Infectious Diseases. 2017;4(3). doi: <https://academic.oup.com/ofid/article/4/3/ofx111/4004900?login=true>. PubMed PMID: WOS:000412357400020.

16. Chan TC, Fan-Ngai Hung I, Ka-Hay Luk J, Chu LW, Hon-Wai Chan F. Effectiveness of Influenza Vaccination in Institutionalized Older Adults: A Systematic Review. Journal of the American Medical Directors Association. 2014;15(3):226.e1-.e6. doi: <http://dx.doi.org/10.1016/j.jamda.2013.10.008>. PubMed PMID: 2014105260.

17. Choi SY, Ha MS, Kim JH, Chi BH, Kim JW, Chang IH, et al. Low-dose versus standard-dose bacille Calmette–Guérin for non-muscle-invasive bladder cancer: Systematic review and meta-analysis of randomized controlled trials. Investigative and Clinical Urology. 2022;63(2):140-50. doi: <http://dx.doi.org/10.4111/icu.20210340>.

18. Clar C, Oseni Z, Flowers N, Keshtkar-Jahromi M, Rees K. Influenza vaccines for preventing cardiovascular disease. Sao Paulo Medical Journal. 2015;133(4):384. doi: <https://doi.org/10.1002/14651858.CD005050.pub3>. PubMed PMID: 606703772.

19. Coelho PLS, Lacerda da Silva Calestini G, Salgueiro Alvo F, de Moura Freitas JM, Vilela Castro PM, Konstantyner T. Safety of human papillomavirus 6, 11, 16 and 18 (recombinant): systematic review and meta-analysis. Revista Paulista de Pediatria. 2015. doi: <https://www.scielo.br/j/rpp/a/3MSXkXzft7QTJg3ZXg8RPbq/?lang=en>.

20. Coleman BL, Sanderson R, Haag MDM, McGovern I. Effectiveness of the MF59-adjuvanted trivalent or quadrivalent seasonal influenza vaccine among adults 65 years of age or older, a systematic review and meta-analysis. Influenza and other Respiratory Viruses. 2021;15(6):813-23. doi: <http://dx.doi.org/10.1111/irv.12871>.

21. Conti A, Broglia G, Sacchi C, Risi F, Barone-Adesi F, Panella M. Efficacy and Safety of Quadrivalent Conjugate Meningococcal Vaccines: A Systematic Review and Meta-Analysis. Vaccines. 2023;11(1). doi: <https://doi.org/10.3390%2Fvaccines11010178>.

22. Corder BN, Bullard BL, Poland GA, Weaver EA. A decade in review: A systematic review of universal influenza vaccines in clinical trials during the 2010 decade. Viruses. 2020;12(10). doi: <http://dx.doi.org/10.3390/v12101186>.

23. Couto E, Sæterdal I, Juvet LK, Klemp M. HPV catch-up vaccination of young women: A systematic review and meta-analysis. BMC Public Health. 2014;14(1). doi: <https://doi.org/10.1186/1471-2458-14-867>.

24. Cuningham W, Geard N, Fielding JE, Braat S, Madhi SA, Nunes MC, et al. Optimal timing of influenza vaccine during pregnancy: A systematic review and meta-analysis. Influenza and other Respiratory Viruses. 2019;13(5):438-52. doi: <https://dx.doi.org/10.1111/irv.12649>. PubMed PMID: 31165580.

25. da Costa VG, Marques-Silva AC, Floriano VG, Moreli ML. Safety, immunogenicity and efficacy of a recombinant tetravalent dengue vaccine: A meta-analysis of randomized trials. Vaccine. 2014;32(39):4885-92. doi: <http://dx.doi.org/10.1016/j.vaccine.2014.07.008>. PubMed PMID: 2014546613.

26. da Silveira LTC, Tura B, Santos M. Systematic review of dengue vaccine efficacy. BMC Infectious Diseases. 2019;19(1):750. doi: <https://dx.doi.org/10.1186/s12879-019-4369-5> PubMed PMID: 31455279.

27. D'Addario M, Redmond S, Scott P, Egli-Gany D, Riveros-Balta AX, Henao Restrepo AM, et al. Two-dose schedules for human papillomavirus vaccine: Systematic review and meta-analysis. Vaccine. 2017;35(22):2892-901. doi: <http://dx.doi.org/10.1016/j.vaccine.2017.03.096>. PubMed PMID: 615676101.

28. Darvishian M, Bijlsma MJ, Hak E, van den Heuvel ER. Effectiveness of seasonal influenza vaccine in community-dwelling elderly people: a meta-analysis of test-negative design case-control studies. The Lancet Infectious Diseases. 2014;14(12):1228-39. doi: <http://dx.doi.org/10.1016/S1473-3099(14)70960-0>. PubMed PMID: 25455990.

29. Darvishian M, Gefenaite G, Turner RM, Pechlivanoglou P, Van Der Hoek W, Van Den Heuvel ER, et al. After adjusting for bias in meta-analysis seasonal influenza vaccine remains effective in community-dwelling elderly. Journal of Clinical Epidemiology. 2014;67(7):734-44. doi: <http://dx.doi.org/10.1016/j.jclinepi.2014.02.009>. PubMed PMID: 2014410405.

30. de Lejarazu-Leonardo RO, Montomoli E, Wojcik R, Christopher S, Mosnier A, Pariani E, et al. Estimation of reduction in influenza vaccine effectiveness due to egg-adaptation changes—systematic literature review and expert consensus. Vaccines. 2021;9(11). doi: <https://doi.org/10.3390/vaccines9111255>.

31. Delere Y, Wichmann O, Klug SJ, van der Sande M, Terhardt M, Zepp F, et al. The efficacy and duration of vaccine protection against human papillomavirus: a systematic review and meta-analysis. Deutsches Ärzteblatt International. 2014;111(35-36):584-91. doi: <https://doi.org/10.3238/arztebl.2014.0584>. PubMed PMID: 25249360; PubMed Central PMCID: PMCPMC4174682.

32. Di Donato V, Caruso G, Petrillo M, Kontopantelis E, Palaia I, Perniola G, et al. Adjuvant hpv vaccination to prevent recurrent cervical dysplasia after surgical treatment: A meta-analysis. Vaccines. 2021;9(5). doi: <https://doi.org/10.3390/vaccines9050410>.

33. Diallo A, Carlos-Bolumbu M, Cervantes-Gonzalez M, Wozniak V, Diallo MH, Diallo BD, et al. Immunogenicity and safety of Ebola virus vaccines in healthy adults: a systematic review and network meta-analysis. Hum Vaccin Immunother. 2021;17(10):3771-83. doi: <https://dx.doi.org/10.1080/21645515.2021.1932214>. PubMed PMID: 34270366.

34. DiazGranados CA, Denis M, Plotkin S. Seasonal influenza vaccine efficacy and its determinants in children and non-elderly adults: A systematic review with meta-analyses of controlled trials. Vaccine. 2012;31(1):49-57. doi: <http://dx.doi.org/10.1016/j.vaccine.2012.10.084>. PubMed PMID: 2012715909.

35. Domnich A, Arata L, Amicizia D, Puig-Barbera J, Gasparini R, Panatto D. Effectiveness of MF59-adjuvanted seasonal influenza vaccine in the elderly: A systematic review and meta-analysis. Vaccine. 2017;35(4):513-20. doi: <https://doi.org/10.1016/j.vaccine.2016.12.011>. PubMed PMID: 614073008.

36. Domnich A, de Waure C. Comparative effectiveness of adjuvanted versus high-dose seasonal influenza vaccines for older adults: a systematic review and meta-analysis. International Journal of Infectious Diseases. 2022;122:855-63. doi: <https://doi.org/10.1016/j.ijid.2022.07.048>.

37. Doyon-Plourde P, Fakih I, Tadount F, Fortin E, Quach C. Impact of influenza vaccination on healthcare utilization - A systematic review. Vaccine. 2019;37(24):3179-89. doi: <https://dx.doi.org/10.1016/j.vaccine.2019.04.051>. PubMed PMID: 31047677.

38. Dzanibe S, Madhi SA. Systematic review of the clinical development of group B streptococcus serotype-specific capsular polysaccharide-based vaccines. Expert Review of Vaccines. 2018;1(7):635-51. doi: <https://www.tandfonline.com/doi/full/10.1080/14760584.2018.1496021>.

39. Ellingson MK, Sheikha H, Nyhan K, Oliveira CR, Niccolai LM. Human papillomavirus vaccine effectiveness by age at vaccination: A systematic review. Hum Vaccin Immunother. 2023;19(2). doi: <https://doi.org/10.1080/21645515.2023.2239085>.

40. Eriksen DO, Jensen PT, Schroll JB, Hammer A. Human papillomavirus vaccination in women undergoing excisional treatment for cervical intraepithelial neoplasia and subsequent risk of recurrence: A systematic review and meta-analysis. Acta Obstetricia et Gynecologica Scandinavica. 2022;101(6):597-607. doi: <https://doi.org/10.1111/aogs.14359>.

41. Faust L, Schreiber Y, Bocking N. A systematic review of BCG vaccination policies among high-risk groups in low TB-burden countries: implications for vaccination strategy in Canadian indigenous communities. BMC Public Health. 2019;19(1):1504. doi: <https://dx.doi.org/10.1186/s12889-019-7868-9>. PubMed PMID: 31711446.

42. Feng S, Cowling BJ, Kelly H, Sullivan SG. Estimating Influenza Vaccine Effectiveness With the Test-Negative Design Using Alternative Control Groups: A Systematic Review and Meta-Analysis. Am J Epidemiol. 2018;187(2):389-97. doi: <https://dx.doi.org/10.1093/aje/kwx251>. PubMed PMID: 28641373.

43. Friedman L, Renaud A, Hines D, Winter A, Bolotin S, Johnstone J, et al. Exploring indirect protection associated with influenza immunization - A systematic review of the literature. Vaccine. 2019;37(49):7213-32. doi: <https://dx.doi.org/10.1016/j.vaccine.2019.09.086>. PubMed PMID: 31648907.

44. Fukuta H, Goto T, Wakami K, Kamiya T, Ohte N. The effect of influenza vaccination on mortality and hospitalization in patients with heart failure: a systematic review and meta-analysis. Heart Fail Rev. 2019;24(1):109-14. doi: <https://dx.doi.org/10.1007/s10741-018-9736-6>. PubMed PMID: 30367316.

45. Garland SM, Kjaer SK, Munoz N, Block SL, Brown DR, DiNubile MJ, et al. Impact and Effectiveness of the Quadrivalent Human Papillomavirus Vaccine: A Systematic Review of 10 Years of Real-world Experience. Clin Infect Dis. 2016;63(4):519-27. doi: <https://dx.doi.org/10.1093/cid/ciw354>. PubMed PMID: 27230391.

46. Gartner BC, Weinke T, Wahle K, Kwetkat A, Beier D, Schmidt KJ, et al. Importance and value of adjuvanted influenza vaccine in the care of older adults from a European perspective - A systematic review of recently published literature on real-world data. Vaccine. 2022;40(22):2999-3008. doi: <https://dx.doi.org/10.1016/j.vaccine.2022.04.019>. PubMed PMID: 35459556.

47. Genovese C, V LAF, Squeri A, Trimarchi G, Squeri R. HPV vaccine and autoimmune diseases: systematic review and meta-analysis of the literature. Journal of Preventive Medicine & Hygiene. 2018;1(3):E194-e9. doi: <https://doi.org/10.15167/2421-4248/jpmh2018.59.3.998>.

48. Goodman E, Reuschenbach M, Kaminski A, Ronnebaum S. Human Papillomavirus Vaccine Impact and Effectiveness in Six High-Risk Populations: A Systematic Literature Review. Vaccines. 2022;10(9). doi: <https://doi.org/10.3390/vaccines10091543>.

49. Gotuzzo E, Yactayo S, Cordova E. Efficacy and duration of immunity after yellow fever vaccination: systematic review on the need for a booster every 10 years. American Journal of Tropical Medicine and Hygiene. 2013;89(3):434-44. doi: <http://www.ncbi.nlm.nih.gov/pubmed/24006295>. PubMed PMID: 24006295.

50. Grassly NC. Immunogenicity and effectiveness of routine immunization with 1 or 2 doses of inactivated poliovirus vaccine: systematic review and meta-analysis. (Special Issue: The final phase of polio eradication and endgame strategies for the post-eradication era.). Journal of Infectious Diseases. 2014;210(Suppl. 1):S439-S46. doi: <http://jid.oxfordjournals.org/content/210/suppl_1/S439.long>. PubMed PMID: 20143413694.

51. Griffiths UK, Clark A, Gessner B, Miners A, Sanderson C, Sedyaningsih ER, et al. Dose-specific efficacy of Haemophilus influenzae type b conjugate vaccines: A systematic review and meta- Analysis of controlled clinical trials. Epidemiology and Infection. 2012;140(8):1343-55. doi: <http://dx.doi.org/10.1017/S0950268812000957>. PubMed PMID: 2012418453.

52. Guo J, Guo S, Dong S. Efficacy, immunogenicity and safety of HPV vaccination in Chinese population: A meta-analysis. Frontiers in Public Health. 2023;11:1128717. doi: <https://doi.org/10.3389/fpubh.2023.1128717>.

53. Hamad MA, Allam H, Sulaiman A, Murali K, Cheikh Hassan HI. Systematic Review and Meta-analysis of Herpes Zoster Vaccine in Patients With CKD. Kidney International Reports. 2021;6(5):1254-64. doi: <http://dx.doi.org/10.1016/j.ekir.2021.02.024>.

54. Harder T, Wichmann O, Klug SJ, van der Sande MAB, Wiese-Posselt M. Efficacy, effectiveness and safety of vaccination against human papillomavirus in males: a systematic review. BMC Medicine. 2018;1(1):110. doi: <https://doi.org/10.1186/s12916-018-1098-3>.

55. Hawken J, Troy SB. Adjuvants and inactivated polio vaccine: A systematic review. Vaccine. 2012;30(49):6971-9. doi: <https://www.ncbi.nlm.nih.gov/pmc/articles/PMC3529007/>. PubMed PMID: 2012666440.

56. Infante V, Miyaji KT, Soarez PC, Sartori AMC. Systematic review and meta-analysis of HPV vaccination in women with systemic lupus erythematosus (SLE). Expert Review of Vaccines. 2021;20(3):309-18. doi: <https://dx.doi.org/10.1080/14760584.2021.1889375>. PubMed PMID: 33573404.

57. Jackson C, Mann A, Mangtani P, Fine P. Effectiveness of haemophilus influenzae type b vaccines administered according to various schedules: Systematic review and meta-analysis of observational data. Pediatric Infectious Disease Journal. 2013;32(11):1261-9. doi: <http://dx.doi.org/10.1097/INF.0b013e3182a14e57>. PubMed PMID: 2013691302.

58. Jones-Gray E, Robinson EJ, Kucharski AJ, Fox A, Sullivan SG. Does repeated influenza vaccination attenuate effectiveness? A systematic review and meta-analysis. Lancet Respir Med. 2023;11(1):27-44. doi: <https://dx.doi.org/10.1016/S2213-2600(22)00266-1>. PubMed PMID: 36152673.

59. Kechagias KS, Kalliala I, Bowden SJ, Athanasiou A, Paraskevaidi M, Paraskevaidis E, et al. Role of human papillomavirus (HPV) vaccination on HPV infection and recurrence of HPV related disease after local surgical treatment: systematic review and meta-analysis. BMJ. 2022. doi: <https://doi.org/10.1136%2Fbmj-2022-070135>.

60. Kling K, Domingo C, Bogdan C, Duffy S, Harder T, Howick J, et al. Duration of protection after vaccination against yellow fever - systematic review and meta-analysis. Clinical Infectious Diseases 2022. doi: <https://doi.org/10.1093/cid/ciac580>.

61. Kuo AM, Brown JN, Clinard V. Effect of influenza vaccination on international normalized ratio during chronic warfarin therapy. Journal of Clinical Pharmacy and Therapeutics. 2012;37(5):505-9. doi: <http://dx.doi.org/10.1111/j.1365-2710.2012.01341.x>. PubMed PMID: 2012525641.

62. Lansbury LE, Smith S, Beyer W, Karamehic E, Pasic-Juhas E, Sikira H, et al. Effectiveness of 2009 pandemic influenza A(H1N1) vaccines: A systematic review and meta-analysis. Vaccine. 2017;35(16):1996-2006. doi: <http://dx.doi.org/10.1016/j.vaccine.2017.02.059>. PubMed PMID: 615074883.

63. LeBras MH, Barry AR. Influenza Vaccination for Secondary Prevention of Cardiovascular Events: A Systematic Review. The Canadian Journal of Hospital Pharmacy. 2017;70(1):27-34. doi: <https://www.ncbi.nlm.nih.gov/pmc/articles/PMC5358055/>. PubMed PMID: MEDLINE:28348430.

64. Lee KR, Bae JH, Hwang IC, Kim KK, Suh HS, Ko KD. Effect of Influenza Vaccination on Risk of Stroke: A Systematic Review and Meta-Analysis. Neuroepidemiology. 2017;48(3-4):103-10. doi: <https://dx.doi.org/10.1159/000478017>. PubMed PMID: 28628919.

65. Lee MD, Lin CH, Lei WT, Chang HY, Lee HC, Yeung CY, et al. Does Vitamin D Deficiency Affect the Immunogenic Responses to Influenza Vaccination? A Systematic Review and Meta-Analysis. Nutrients. 2018;10(4):26. doi: <https://dx.doi.org/10.3390/nu10040409>. PubMed PMID: 29587438.

66. Lee JKH, Lam GKL, Shin T, Samson SI, Greenberg DP, Chit A. Efficacy and effectiveness of high-dose influenza vaccine in older adults by circulating strain and antigenic match: An updated systematic review and meta-analysis. Vaccine. 2021;39 Suppl 1:A24-A35. doi: <https://dx.doi.org/10.1016/j.vaccine.2020.09.004>. PubMed PMID: 33422382.

67. Lei WT, Shih PC, Liu SJ, Lin CY, Yeh TL. Effect of Probiotics and Prebiotics on Immune Response to Influenza Vaccination in Adults: A Systematic Review and Meta-Analysis of Randomized Controlled Trials. Nutrients. 2017;9(11):27. doi: <https://dx.doi.org/10.3390/nu9111175>. PubMed PMID: 29077061.

68. Leung VK, Cowling BJ, Feng S, Sullivan SG. Concordance of interim and final estimates of influenza vaccine effectiveness: A systematic review. Eurosurveillance. 2016;21(16). doi: <http://dx.doi.org/10.2807/1560-7917.ES.2016.21.16.30202>. PubMed PMID: 610201205.

69. Leung J, Broder KR, Marin M. Severe varicella in persons vaccinated with varicella vaccine (breakthrough varicella): a systematic literature review. Expert Review of Vaccines. 2017;16(4):391-400. doi: <https://www.ncbi.nlm.nih.gov/pmc/articles/PMC5544348/>. PubMed PMID: 20173127552.

70. Li T, Qi X, Li Q, Tang W, Su K, Jia M, et al. A systematic review and meta-analysis of seasonal influenza vaccination of health workers. Vaccines. 2021;9(10). doi: <https://doi.org/10.3390/vaccines9101104>.

71. Lichter K, Krause D, Xu J, Tsai SHL, Hage C, Weston E, et al. Adjuvant Human Papillomavirus Vaccine to Reduce Recurrent Cervical Dysplasia in Unvaccinated Women: A Systematic Review and Meta-analysis. Obstetrics and Gynecology. 2020;135(5):1070-83. doi: <https://doi.org/10.1097/aog.0000000000003833>.

72. Lu B, Kumar A, Castellsague X, Giuliano AR. Efficacy and Safety of Prophylactic Vaccines against Cervical HPV Infection and Diseases among Women: A Systematic Review & Meta-Analysis. BMC Infectious Diseases. 2011;11(13). doi: <http://dx.doi.org/10.1186/1471-2334-11-13>. PubMed PMID: 2011077270.

73. Lukacs A, Mate Z, Farkas N, Miko A, Tenk J, Hegyi P, et al. The quadrivalent HPV vaccine is protective against genital warts: a meta-analysis. BMC Public Health. 2020;20(1):691. doi: <https://dx.doi.org/10.1186/s12889-020-08753-y>. PubMed PMID: 32460747.

74. Mac Eochagain C, Power R, Parker I, Brennan D. HPV vaccination among seropositive, DNA negative cohorts: a systematic review & meta-analysis. J. 2022;33(3):e24. doi: <https://dx.doi.org/10.3802/jgo.2022.33.e24>. PubMed PMID: 35128855.

75. Malagón T, Drolet M, Boily MC, Franco EL, Jit M, Brisson J, et al. Cross-protective efficacy of two human papillomavirus vaccines: A systematic review and meta-analysis. The Lancet Infectious Diseases. 2012;12(10):781-9. doi: <https://doi.org/10.1016/S1473-3099(12)70187-1>.

76. Malisheni M, Khaiboullina SF, Rizvanov AA, Takah N, Murewanhema G, Bates M. Clinical efficacy, safety, and immunogenicity of a live attenuated tetravalent dengue vaccine (CYD-TDV) in children: A systematic review with meta-analysis. Frontiers in Immunology. 2017;8 (AUG) (no pagination)(863). doi: <http://dx.doi.org/10.3389/fimmu.2017.00863>. PubMed PMID: 617629200.

77. Markowitz LE, Drolet M, Perez N, Jit M, Brisson M. Human papillomavirus vaccine effectiveness by number of doses: Systematic review of data from national immunization programs. [Review]. Vaccine. 2018;1(32 Pt A):4806-15. doi: <https://doi.org/10.1016/j.vaccine.2018.01.057>.

78. Markowitz LE, Drolet M, Lewis RM, Lemieux-Mellouki P, Pérez N, Jit M, et al. Human papillomavirus vaccine effectiveness by number of doses: Updated systematic review of data from national immunization programs. Vaccine. 2022. doi: <https://doi.org/10.1016/j.vaccine.2022.06.065>.

79. Mavundza EJ, Wiyeh AB, Mahasha PW, Halle-Ekane G, Wiysonge CS. A systematic review of immunogenicity, clinical efficacy and safety of human papillomavirus vaccines in people living with the human immunodeficiency virus. Hum Vaccin Immunother. 2020;16(2):426-35. doi: <https://dx.doi.org/10.1080/21645515.2019.1656481>. PubMed PMID: 31448991.

80. McGirr A, Widenmaier R, Curran D, Espie E, Mrkvan T, Oostvogels L, et al. The comparative efficacy and safety of herpes zoster vaccines: A network meta-analysis. Vaccine. 2019;37(22):2896-909. doi: <https://dx.doi.org/10.1016/j.vaccine.2019.04.014>. PubMed PMID: 30982636.

81. Miazga W, Wnuk K, Tatara T, Świtalski J, Matera A, Religioni U, et al. The long-term efficacy of tick-borne encephalitis vaccines available in Europe - a systematic review. BMC Infectious Diseases. 2023;23(1). doi: <https://doi.org/10.1186/s12879-023-08562-9>.

82. Montagnani C, Chiappini E, Galli L, de Martino M. Vaccine against tuberculosis: What's new? BMC Infectious Diseases. 2014;14 (no pagination)(S2). doi: <http://dx.doi.org/10.1186/1471-2334-14-S1-S2>. PubMed PMID: 612366519.

83. Mousavi T, Moosazadeh M, Afshari M, Davoodi L, Haghshenas M. Efficacy of L1 protein vaccines against cervical and vaginal cancer: A systematic review and meta-analysis. Iranian Red Crescent Medical Journal. 2016;19 (4) (no pagination)(e42732). doi: <https://www.researchgate.net/profile/Tahoora-Mousavi-2/publication/311626109_Efficacy_of_L1_Protein_Vaccines_Against_Cervical_and_Vaginal_Cancer_A_Systematic_Review_and_Meta-Analysis/links/587f279f08ae9275d4ebae91/Efficacy-of-L1-Protein-Vaccines-Against-Cervical-and-Vaginal-Cancer-A-Systematic-Review-and-Meta-Analysis.pdf>. PubMed PMID: 615854882.

84. Nielsen KJ, Jakobsen KK, Jensen JS, Gronhoj C, Von Buchwald C. The Effect of Prophylactic HPV Vaccines on Oral and Oropharyngeal HPV Infection-A Systematic Review. Viruses. 2021;13(7):11. doi: <https://dx.doi.org/10.3390/v13071339>. PubMed PMID: 34372545.

85. Ott JJ, Irving G, Wiersma ST. Long-term protective effects of hepatitis A vaccines. A systematic review. Vaccine. 2012;31(1):3-11. doi: <http://dx.doi.org/10.1016/j.vaccine.2012.04.104>. PubMed PMID: 2012715902.

86. Pan Y, Du L, Gan Q, Ma W, Wang M, Lu Z, et al. Meta-analysis of whether influenza vaccination attenuates symptom severity in vaccinated influenza patients. Public Health Nursing. 2022;39(2):509-16. doi: <https://doi.org/10.1111/phn.12985>.

87. Pittet LF, Curtis N. Does bacillus Calmette-Guerin vaccine prevent herpes simplex virus recurrences? A systematic review. Reviews in Medical Virology. 2020;31(1):1-9. doi: <https://europepmc.org/article/med/32975011>. PubMed PMID: 32975011.

88. Pittet LF, Thøstesen LM, Aaby P, Kofoed PE, Curtis N, Benn CS. Neonatal Bacillus Calmette-Guérin Vaccination to Prevent Early-Life Eczema: A Systematic Review and Meta-analysis. Dermatitis. 2022. doi: <https://www.ncbi.nlm.nih.gov/pmc/articles/PMC9674447/>.

89. Ponduri A, Azmy MC, Axler E, Lin J, Schwartz R, Chirilă M, et al. The Efficacy of Human Papillomavirus Vaccination as an Adjuvant Therapy in Recurrent Respiratory Papillomatosis. Laryngoscope. 2023. doi: <https://doi.org/10.1002/lary.30560>.

90. Prandi GM, Cocchio S, Fonzo M, Furlan P, Nicoletti M, Baldo V. Towards the elimination of cervical cancer: HPV epidemiology, real-world experiences and the potential impact of the 9-valent HPV vaccine. European Journal of Gynaecological Oncology. 2021;42(5):1068-78. doi: <http://doi.org/10.31083/j.ejgo4205156>.

91. Preiss S, Chanthavanich P, Chen LH, Marano C, Buchy P, van Hoorn R, et al. Post-exposure prophylaxis (PEP) for rabies with purified chick embryo cell vaccine: a systematic literature review and meta-analysis. Expert Review of Vaccines. 2018;1(6):525-45. doi: <https://doi.org/10.1080/14760584.2018.1473765>.

92. Puig-Barbera J, Tamames-Gomez S, Plans-Rubio P, Eiros-Bouza JM. Relative Effectiveness of Cell-Cultured versus Egg-Based Seasonal Influenza Vaccines in Preventing Influenza-Related Outcomes in Subjects 18 Years Old or Older: A Systematic Review and Meta-Analysis. Int J Environ Res Public Health. 2022;19(2):12. doi: <https://dx.doi.org/10.3390/ijerph19020818>. PubMed PMID: 35055642.

93. Rampa JE, Askling HH, Lang P, Zens KD, Gultekin N, Stanga Z, et al. Immunogenicity and safety of the tick-borne encephalitis vaccination (2009-2019): A systematic review. Travel Med Infect Dis. 2020;37:101876. doi: <https://dx.doi.org/10.1016/j.tmaid.2020.101876>. PubMed PMID: 32931931.

94. Ren X, Hao Y, Wu B, Jia X, Niu M, Wang K, et al. Efficacy of prophylactic human papillomavirus vaccines on cervical cancer among the Asian population: A meta-analysis. Frontiers in Microbiology. 2022;13. doi: <https://doi.org/10.3389/fmicb.2022.1052324>.

95. Rey-Ares L, Ciapponi A, Pichon-Riviere A. Efficacy and safety of human papilloma virus vaccine in cervical cancer prevention: systematic review and meta-analysis. Arch Argent Pediatr. 2012;110(6):483-9. doi: <https://doi.org/10.5546/aap.2012.eng.483>. PubMed PMID: 23224305.

96. Rosado C, Fernandes ÂR, Rodrigues AG, Lisboa C. Impact of Human Papillomavirus Vaccination on Male Disease: A Systematic Review. Vaccines. 2023;11(6). doi: <https://doi.org/10.3390/vaccines11061083>.

97. Sangar VC, Ghongane BB, Mathur G, Chowdhary AS. Safety and adverse events of prophylactic HPV vaccines among healthy women: A systematic review & meta analysis. International Journal of Pharmaceutical Sciences and Research. 2015;6(4):1779-91. doi: <http://dx.doi.org/10.13040/IJPSR.0975-8232.6%284%29.1779-91>. PubMed PMID: 2015896053.

98. Schwerdtle P, Onekon CK, Recoche K. A Quantitative Systematic Review and Meta-Analysis of the Effectiveness of Oral Cholera Vaccine as a Reactive Measure in Cholera Outbreaks. Prehospital Disaster Med. 2018;33(1):2-6. doi: <https://dx.doi.org/10.1017/S1049023X17007166>. PubMed PMID: 29317005.

99. Silverii GA, Clerico A, Fornengo R, Gabutti G, Sordi V, Peruzzi O, et al. Efficacy and effectiveness of Herpes zoster vaccination in adults with diabetes mellitus: a systematic review and meta-analysis of clinical trials and observational studies. Acta Diabetologica. 2023;60(10):1343-9. doi: <https://doi.org/10.1007/s00592-023-02127-7>.

100. Staadegaard L, Rönn MM, Soni N, Bellerose ME, Bloem P, Brisson M, et al. Immunogenicity, safety, and efficacy of the HPV vaccines among people living with HIV: A systematic review and meta-analysis. eClinicalMedicine. 2022;52. doi: <https://doi.org/10.1016%2Fj.eclinm.2022.101585>.

101. Steffen R, Erber W, Schmitt HJ. Can the booster interval for the tick-borne encephalitis (TBE) vaccine 'FSME-IMMUN' be prolonged? - A systematic review. Ticks Tick Borne Dis. 2021;12(5):101779. doi: <https://dx.doi.org/10.1016/j.ttbdis.2021.101779>. PubMed PMID: 34298356.

102. Stuurman AL, Marano C, Bunge EM, De Moerlooze L, Shouval D. Impact of universal mass vaccination with monovalent inactivated hepatitis A vaccines-A systematic review. Hum Vaccin Immunother. 2017;13(3):724-36. doi: <http://dx.doi.org/10.1080/21645515.2016.1242539>. PubMed PMID: 614876590.

103. Tejada RA, Vargas KG, Benites-Zapata V, Mezones-Holguin E, Bolanos-Diaz R, Hernandez AV. Human papillomavirus vaccine efficacy in the prevention of anogenital warts: systematic review and meta-analysis. Salud Publica de Mexico. 2017;59(1):84-94. doi: <https://europepmc.org/article/med/28423114>. PubMed PMID: 20173155935.

104. Teo E, Lockhart K, Purchuri Sai N, Pushparajah J, Cripps Allan W, van Driel Mieke L. Haemophilus influenzae oral vaccination for preventing acute exacerbations of chronic bronchitis and chronic obstructive pulmonary disease. Cochrane Database Syst Rev. 2017;(6). doi: <https://doi.org/10.1002/14651858.CD010010.pub3>. PubMed PMID: CD010010.

105. Truelove SA, Keegan LT, Moss WJ, Chaisson LH, Macher E, Azman AS, et al. Clinical and epidemiological aspects of diphtheria: A systematic review and pooled analysis. Clin Infect Dis. 2020;71(1):89-97. doi: <https://doi.org/10.1093/cid/ciz808>.

106. Ventura C, Luís Â, Soares CP, Venuti A, Paolini F, Pereira L, et al. The Effectiveness of Therapeutic Vaccines for the Treatment of Cervical Intraepithelial Neoplasia 3: A Systematic Review and Meta-Analysis. Vaccines. 2022;10(9). doi: <https://doi.org/10.3390/vaccines10091560>.

107. Wang H, Hu Y, Zhang G, Zheng J, Li L, An Z. Meta-analysis of vaccine effectiveness of mumps-containing vaccine under different immunization srategies in China. Vaccine. 2014;32(37):4806-12. doi: <http://dx.doi.org/10.1016/j.vaccine.2014.05.061>. PubMed PMID: 2014515399.

108. Wang W, Kothari S, Baay M, Garland SM, Giuliano AR, Nygard M, et al. Real-world impact and effectiveness assessment of the quadrivalent HPV vaccine: a systematic review of study designs and data sources. Expert Review of Vaccines. 2021;21(2):227-40. doi: <https://dx.doi.org/10.1080/14760584.2022.2008243>. PubMed PMID: 34845951.

109. Wang WV, Kothari S, Skufca J, Giuliano AR, Sundstrom K, Nygard M, et al. Real-world impact and effectiveness of the quadrivalent HPV vaccine: an updated systematic literature review. Expert Review of Vaccines. 2022;21(12):1799-817. doi: <https://dx.doi.org/10.1080/14760584.2022.2129615>. PubMed PMID: 36178094.

110. Wei F, Alberts CJ, Albuquerque A, Clifford GM. Impact of human papillomavirus vaccine against anal human papillomavirus infection, anal intraepithelial neoplasia, and recurrence of anal intraepithelial neoplasia: a systematic review and meta-analysis. Journal of Infectious Diseases. 2023. doi: <https://doi.org/10.1093/infdis/jiad183>.

111. Whitworth HS, Gallagher KE, Howard N, Mounier-Jack S, Mbwanji G, Kreimer AR, et al. Efficacy and immunogenicity of a single dose of human papillomavirus vaccine compared to no vaccination or standard three and two-dose vaccination regimens: A systematic review of evidence from clinical trials. Vaccine. 2020;38(6):1302-14. doi: <https://dx.doi.org/10.1016/j.vaccine.2019.12.017>. PubMed PMID: 31870572.

112. Wilson L, Gracie L, Kidy F, Thomas GN, Nirantharakumar K, Greenfield S, et al. Safety and efficacy of tuberculosis vaccine candidates in low- and middle-income countries: a systematic review of randomised controlled clinical trials. BMC Infectious Diseases. 2023;23(1):120. doi: <https://dx.doi.org/10.1186/s12879-023-08092-4>.

113. Yeasmin M, Molla MMA, Masud HMAA, Saif-Ur-Rahman KM. Safety and immunogenicity of Zika virus vaccine: A systematic review of clinical trials. Reviews in Medical Virology. 2022. doi: <https://doi.org/10.1002/rmv.2385>.

114. Yedlapati SH, Khan SU, Talluri S, Lone AN, Khan MZ, Khan MS, et al. Effects of Influenza Vaccine on Mortality and Cardiovascular Outcomes in Patients With Cardiovascular Disease: A Systematic Review and Meta-Analysis. J Am Heart Assoc. 2021;10(6):e019636. doi: <https://dx.doi.org/10.1161/JAHA.120.019636>. PubMed PMID: 33719496.

115. Yin M, Xu X, Liang Y, Ni J. Effectiveness, immunogenicity and safety of one vs. two-dose varicella vaccination:a meta-analysis. Expert Review of Vaccines. 2018;17(4):351-62. doi: <https://dx.doi.org/10.1080/14760584.2018.1433999>. PubMed PMID: 29388450.

116. Zeevaert R, Thiry N, Maertens de Noordhout C, Roberfroid D. Efficacy and safety of the recombinant zoster vaccine: A systematic review and meta-analysis. Vaccine: X. 2023;15. doi: <https://doi.org/10.1016/j.jvacx.2023.100397>.

117. Zhang Y, Zhang H, Wang B, Song G, Hayden JC, Amirthalingam P, et al. Pregnancy outcomes after a mass vaccination campaign with an oral cholera vaccine: a systematic review and meta-analysis. Bjog. 2020;127(9):1066-73. doi: <https://dx.doi.org/10.1111/1471-0528.16260>. PubMed PMID: 32289871.

118. Zhang J, Qin Z, Lou C, Huang J, Xiong Y. The efficacy of vaccination to prevent human papilloma viruses infection at anal and oral: a systematic review and meta-analysis. Public Health. 2021;196:165-71. doi: <https://dx.doi.org/10.1016/j.puhe.2021.05.012>. PubMed PMID: 34229128.

119. Zhang Z, Suo L, Pan J, Zhao D, Lu L. Two-dose varicella vaccine effectiveness in China: a meta-analysis and evidence quality assessment. BMC Infectious Diseases. 2021;21(1):543. doi: <https://dx.doi.org/10.1186/s12879-021-06217-1>. PubMed PMID: 34107891.

120. Zizza A, Banchelli F, Guido M, Marotta C, Di Gennaro F, Mazzucco W, et al. Efficacy and safety of human papillomavirus vaccination in HIV-infected patients: a systematic review and meta-analysis. Sci. 2021;11(1):4954. doi: <https://dx.doi.org/10.1038/s41598-021-83727-7>. PubMed PMID: 33654181.
